# Black Phosphorus Accelerates Bone Regeneration Based on Immunoregulation

**DOI:** 10.1002/advs.202304824

**Published:** 2023-11-12

**Authors:** Minglong Qiu, Nijiati Tulufu, Guoqing Tang, Wenkai Ye, Jin Qi, Lianfu Deng, Changwei Li

**Affiliations:** ^1^ Department of Orthopaedics Shanghai Key Laboratory for Prevention and Treatment of Bone and Joint Diseases Shanghai Institute of Traumatology and Orthopaedics Ruijin Hospital Shanghai Jiao Tong University School of Medicine 197 Ruijin 2nd Road Shanghai 200025 P. R. China; ^2^ Kunshan Hospital of Traditional Chinese Medicine Affiliated Hospital of Yangzhou University 388 Zuchongzhi Road Kunshan City Jiangsu Province 215300 P. R. China

**Keywords:** black phosphorus, bone regeneration, gene knocking‐out, immunoregulation, RNA sequencing

## Abstract

A fundamental understanding of inflammation and tissue healing suggests that the precise regulation of the inflammatory phase, both in terms of location and timing, is crucial for bone regeneration. However, achieving the activation of early inflammation without causing chronic inflammation while facilitating quick inflammation regression to promote bone regeneration continues to pose challenges. This study reveals that black phosphorus (BP) accelerates bone regeneration by building an osteogenic immunological microenvironment. BP amplifies the acute pro‐inflammatory response and promotes the secretion of anti‐inflammatory factors to accelerate inflammation regression and tissue regeneration. Mechanistically, BP creates an osteoimmune‐friendly microenvironment by stimulating macrophages to express interleukin 33 (IL‐33), amplifying the inflammatory response at an early stage, and promoting the regression of inflammation. In addition, BP‐mediated IL‐33 expression directly promotes osteogenic differentiation of bone marrow mesenchymal stem cells (BMSCs), which further facilitates bone repair. To the knowledge, this is the first study to reveal the immunomodulatory potential of BP in bone regeneration through the regulation of both early‐stage inflammatory responses and later‐stage inflammation resolution, along with the associated molecular mechanisms. This discovery serves as a foundation for the clinical use of BP and is an efficient approach for managing the immune microenvironment during bone regeneration.

## Graphical abstract

This study provides an immunomodulatory strategy to promote bone regeneration. Black phosphorus (BP) creates an osteoimmune‐friendly microenvironment by stimulating macrophages to express interleukin 33 (IL‐33), amplifying the inflammatory response at an early stage and promoting the regression of inflammation. In addition, BP‐mediated IL‐33 expression directly promotes the osteogenic differentiation of bone marrow mesenchymal stem cells (BMSCs), further facilitating bone repair.

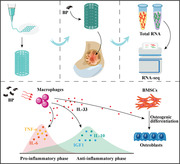

## 1 Introduction

Natural bone healing resembles the process of bone development and involves multiple physiological events, including hematoma formation, inflammatory responses, recruitment of progenitor cells for cartilage production, vascular regeneration and calcification, and bone remodeling.^[^ ^1^ ^]^ The inflammatory response is the initial stage that almost all types of bone defects need to undergo and consists of an early acute pro‐inflammatory response and later remission of inflammation, mainly to recruit cells, remove necrotic tissue, secrete cytokines and growth factors, and promote cell differentiation.^[^ ^2^ ^]^ The current understanding of the aseptic inflammatory response in bone repair and the design of biomaterials for bone repair is primarily concerned with suppressing the acute pro‐inflammatory response and promoting the subsequent anti‐inflammatory phase.^[^ ^3^ ^]^ However, with an increased understanding of the immune regulation of bone repair, the suppression of early acute inflammation is considered detrimental to bone healing without other comorbid trauma and infection.^[^ ^4^ ^]^

During the acute inflammatory phase, monocytes differentiate into classically activated pro‐inflammatory (M1) macrophages, which form a pro‐inflammatory microenvironment rich in cytokines, such as interleukin 1 (IL‐1), interleukin 6 (IL‐6), and tumor necrosis factor (TNF).^[^ ^5^ ^]^ The regression of inflammation is mainly driven by alternatively activated anti‐inflammatory (M2) macrophages, regulatory T cells (Tregs), and helper T cell 2 (Th2) cells.^[^ ^6^ ^]^ Interleukin 10 (IL‐10), transforming growth factor‐beta (TGF‐*β*), and insulin‐like growth factor 1 (IGF‐1) are critical anti‐inflammatory cytokines.^[^ ^7^ ^]^ The acute pro‐inflammatory and anti‐inflammatory phases are neither independent nor antagonistic to each other, and many of the recognized pro‐inflammatory cytokines produced during the acute pro‐inflammatory phase, such as interleukin 42 (IL‐42) and IL‐6, contribute to the regression of inflammation.^[^ ^8^ ^]^ The acute proinflammatory phase prepares for the anti‐inflammatory phase and creates an immune microenvironment conducive to bone regeneration.^[^ ^4^ ^]^ The depletion of M1 macrophages results in a marked decrease in cytokines, such as IL‐6 and TNF, leading to impaired fracture healing.^[^ ^9^ ^]^ The well‐known non‐steroidal anti‐inflammatory drugs can increase the risk of bone nonhealing.^[^ ^10^ ^]^ In contrast, the sequential activation of pro‐ and anti‐inflammatory agents promotes bone regeneration.^[^ ^11^ ^]^ Therefore, the design of bone repair biomaterials should also emphasize the appropriate activation of the acute inflammatory response to indirectly enhance osteogenesis, thus contributing to the modulation of a favorable bone immune microenvironment.^[^ ^11^, ^12^ ^]^

Over the past decade, various biological materials have been used to modulate the immune microenvironment during bone regeneration.^[^ ^13^ ^]^ These include altering the physicochemical properties and surface morphology of the material, loading anti‐inflammatory agents or cytokines and metal ions, and modulating the immune response using the extracellular matrix or cells to alter the macrophage phenotype induced after implantation.^[^ ^4^, ^14^ ^]^ However, consistent with the perception of bone immunomodulation, most biomaterials focus on suppressing acute inflammation and promoting macrophage polarization, ignoring the importance of the acute pro‐inflammatory phase in the overall bone repair process.^[^ ^4^, ^15^ ^]^ The ideal bone immunomodulatory biomaterial should appropriately activate acute inflammation to create an immune microenvironment conducive to bone regeneration without leading to long‐term chronic inflammation, which manifests as the inhibition of bone regeneration.^[^ ^4^ ^]^ However, most biomaterials produce a complex inflammatory response that is difficult to manipulate after implantation and may inhibit bone regeneration owing to the slow degradation of biomaterials, which may produce a persistent chronic inflammatory response.^[^ ^16^ ^]^ Therefore, biomaterials that can adequately activate the early inflammatory response and promote the regression of subsequent inflammation are required to accelerate bone regeneration.

The use of biological materials based on their biological constituents is a promising strategy.^[^ ^13^ ^]^ However, most natural organic polymers, such as collagen, fibrin, gelatin, sodium alginate, cellulose, and hyaluronic acid, exhibit anti‐inflammatory properties.^[^ ^16^ ^]^ Hydroxyapatite and calcium phosphate have been shown to have pro‐inflammatory properties, promoting the expression of pro‐inflammatory factors, such as IL‐1 and IL‐6, and also act as vaccine adjuvants to enhance the immune response.^[^ ^17^ ^]^ However, their slow degradation rates may lead to chronic inflammation.^[^ ^18^ ^]^ Recently, black phosphorus (BP), which is composed of a single phosphorus element with a unique laminar structure, has demonstrated significant potential in regenerative medicine, primarily because of its excellent biocompatibility and continuous release of phosphate ions during oxidation.^[^ ^19^ ^]^ Phosphate is a fundamental component that forms the phosphate backbone in DNA and RNA, and the phospholipid bilayer in cell membranes; thus, it plays an indispensable role in cell signaling, proliferation, and differentiation.^[^ ^20^ ^]^ A sufficient supply of phosphate ions is crucial for skeletal development, cell growth, and osteogenesis during the repair of bone injuries.^[^ ^21^ ^]^ After oxidation, BP transforms into nontoxic phosphate, which attracts the surrounding free calcium ions and binds them to form calcium phosphate. This process stimulates mineralization and deposition, which are crucial for in situ bone regeneration and repair.^[^ ^22^ ^]^ Additionally, BP‐based nanomaterials possess a stable and undamaged release mechanism that can be controlled by light owing to their strong light absorption ability in the NIR region. This characteristic ensures a more stable and long‐lasting release of loaded drugs.^[^ ^23^ ^]^ Moreover, local hyperthermia treatment using BP has been shown to upregulate alkaline phosphatase (ALP) and heat shock protein (HSP), resulting in an increased formation of mineralized crystals and facilitation of longitudinal and concentric bone growth.^[^ ^24^ ^]^ Thus, the high biocompatibility, biodegradability, and controlled‐release properties of BP‐based nanomaterials make them highly promising candidates for further advancement in the field of bone repair.

Not only does BP supply phosphate, it has also been noted to have potential inflammatory effects. It has been observed that the intravenous injection of bare BP nanosheets induces inflammatory responses in mice and macrophages.^[^ ^25^ ^]^ Notably, the inflammatory responses induced by the BP nanosheets are reversible, with acute inflammation recovering quickly to normal levels. This suggests that BP may also play a role in the resolution of inflammation and not just in promoting acute inflammation.^[^ ^25a^ ^]^ The interaction between BMSCs and macrophages is crucial for successful bone healing.^[^ ^26^ ^]^ When murine BMSCs were co‐cultured with M1 macrophages, it enhanced bone mineralization and alkaline phosphatase (ALP) activity. Additionally, adding interleukin 4 (IL‐4) to polarize M1 macrophages to the M2 phenotype further increased the formation of a calcified matrix.^[^ ^27^ ^]^ Therefore, scaffolds based on BP immunofunctionalisation, such as those coated with IL‐4, have been utilized to improve bone regeneration.^[^ ^19b^ ^]^ Although these three dimensional (3D) immune scaffolds have demonstrated promising results in the treatment of bone repair, it is crucial to acknowledge that their immunoregulatory function primarily relies on the adsorption of immunomodulatory cytokines, such as IL‐4. However, the potential regulatory effects of BP nanosheets on inflammation during bone repair remain unclear. Notably, Long et al. reported that poly (lactic‐co‐glycolic acid) (PLGA)/BP scaffolds were extremely effective in inhibiting inflammation by converting macrophages from the M1 to M2 phenotype, which in turn promoted bone regeneration in the distal femoral defect region of steroid‐associated osteonecrosis.^[^ ^28^ ^]^ However, it is still unknown whether BP can activate early inflammatory responses during bone repair. Given the significant positive impact of early acute inflammation and the subsequent resolution of inflammation on bone healing, it is imperative to conduct further research to understand the regulatory role of BP in the inflammatory phase. This included examining the influence of BP on both initiation and resolution of inflammation during the bone repair process, as well as exploring the underlying molecular mechanisms at play.

In this study, a polycaprolactone‐BP (PCP) 3D scaffold was constructed by loading BP onto a 3D‐printed polycaprolactone (PCL) scaffold. The results revealed that the PCP scaffold significantly accelerated bone regeneration compared with the PCL scaffold. Moreover, our data revealed that PCP accelerated bone regeneration by creating an osteogenic immunological microenvironment by stimulating the expression of early pro‐inflammatory factors IL‐6 and TNF‐*α* to enhance the pro‐inflammatory phase and also by stimulating the expression of anti‐inflammatory factors IL‐10 and IGF‐1 to facilitate inflammation regression in the subsequent stages. Mechanistically, BP creates an osteoimmune‐friendly microenvironment by stimulating macrophages to express interleukin 33 (IL‐33), thereby amplifying the inflammatory response in the early stages and promoting inflammation regression later. Additionally, BP‐mediated IL‐33 expression directly promotes osteogenic differentiation of BMSCs, further facilitating bone repair. In conclusion, this study provides evidence that BP accelerates bone regeneration by modulating immune responses at the site of injury and creating a microenvironment conducive to regeneration.

## 2 Results and Discussion

### 2.1 Fabrication and Characterization of PCP Scaffold

**Figure 1:**
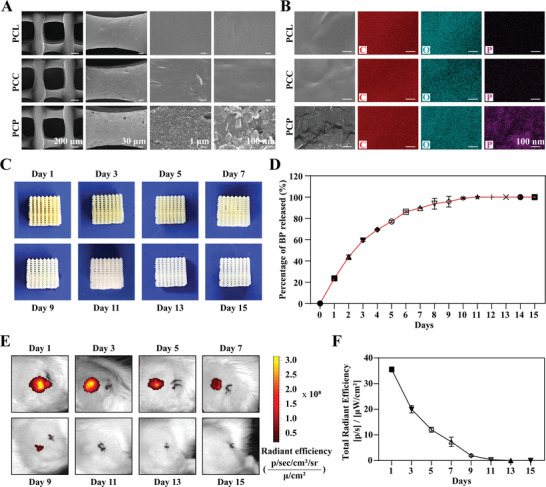
Characterization of the PCP scaffold. A) SEM images of PCL, PCC, and PCP scaffolds. B) SEM‐EDS images of the PCL, PCC, and PCP scaffolds. C) Images of PCP scaffold release in water. D) Release curves of PCP scaffolds in water. E) Images of PCP scaffold release in vivo. F) Release curves of PCP scaffolds in vivo.

**Scheme 1:**
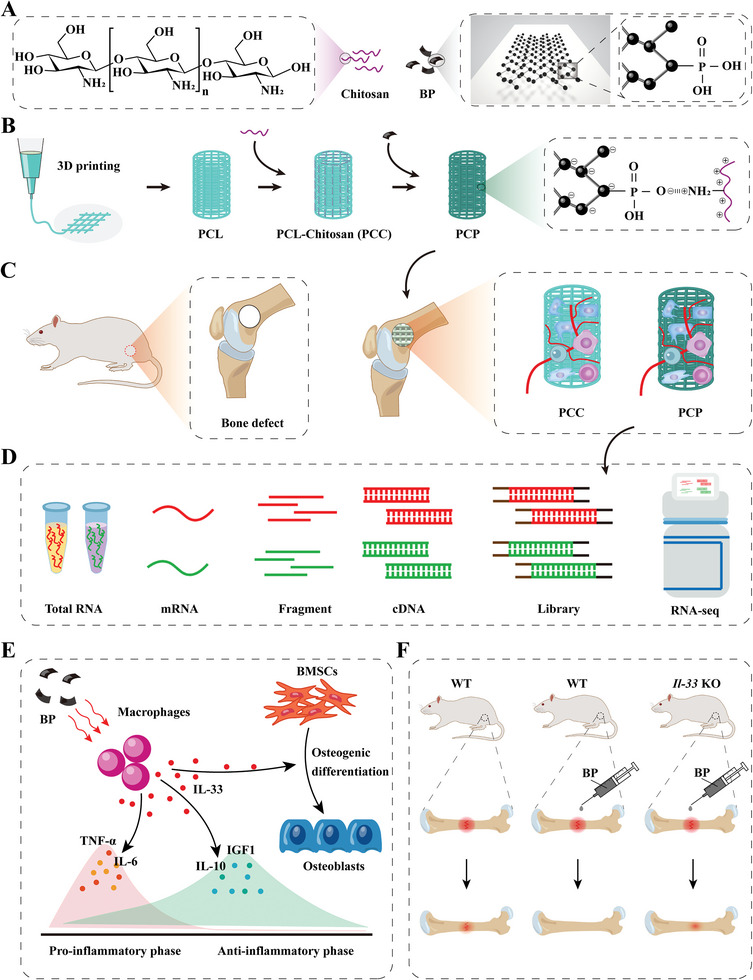
Schematic diagram of the fabrication, implantation, RNA‐seq, and regulation of bone regeneration of scaffolds loaded with BP. A) Schematic diagram of the molecular structure of chitosan and the two‐dimensional structure of BP, with the dashed box on the right showing the phosphate formed after oxidation of BP. B) Schematic diagram of PCP scaffold fabrication. C) Schematic diagram of the construction and scaffold implantation of a bone defect model in the rat femur. D) Schematic diagram of RNA‐seq. E) Schematic diagram of BP accelerates bone regeneration by stimulating the expression of IL‐33 in macrophages. F) The potential of BP to accelerate bone repair was reduced after *Il‐33* knockout. BP, black phosphorus. PCL, polycaprolactone scaffold. PCC, polycaprolactone‐chitosan scaffold. PCP, polycaprolactone‐chitosan‐black phosphorus scaffold. RNA‐seq, whole transcriptome sequencing. mRNA, messenger RNA. cDNA, complementary DNA.

To facilitate the subsequent acquisition of biomaterials with tissue cells for RNA sequencing (RNA‐seq), we constructed 3D scaffolds using 3D printing technology, as 3D scaffolds facilitate the invasion and local stimulation of tissue cells. PCL has a low melting point (favorable for molding during manufacturing) and suitable properties for bone‐tissue regeneration.^[^ ^29^ ^]^ They can be fabricated into desired porous structures via 3D printing.^[^ ^30^ ^]^ A PCP scaffold was first constructed using 3D printing technology. The printed filament diameter was 200 µm, and the filament pitch was 400 µm. Consistent with previous findings, a filament pitch with 100–1200 µm is ideal for bone regeneration.^[^ ^31^ ^]^ The liquefier temperature was set to 90 °C for the melt of PCL.^[^ ^32^ ^]^ The PCL scaffold was fabricated using a 0/90° laminated pattern of cellular lattice structures and continuous contour filaments to ensure a 3D porous spatial structure (**Figure** **1**A). To load BP nanosheets (Figure S1, Supporting Information) onto the 3D PCL scaffold, we used surface alkaline etching treatment and *N*‐(3‐Dimethylaminopropyl)‐*N^′^*‐ethylcarbodiimide hydrochloride (EDC)/*N*‐Hydroxysuccinimide (NHS) activation to covalently graft positively charged chitosan onto the surface of the 3D PCL scaffold, forming irregular pores, and efficiently loading BP nanosheets through electrostatic interactions (**Scheme** **1**A,B). Scanning electron microscopy (SEM) showed that BP was successfully loaded onto the surface of the PCL scaffold. The magnified image on the right clearly depicts the BP (Figure 1A). Using SEM combined with energy‐dispersive X‐ray spectroscopy (SEM‐EDS), we further mapped the elements on the surface of the PCP scaffolds and discovered that the elements carbon (C, red) and oxygen (O, green) were evenly distributed within the frameworks (Figure 1B), while phosphorus elements (P, purple) were not detected on the PCL and PCL‐chitosan (PCC) scaffold, but were clearly observed on the PCP scaffold, constituting ≈1.41% of the total elemental composition (Figure S2A, Supporting Information). Consistently, surface energy spectroscopy analysis showed the characteristic P‐element peak spectra on the surface of the PCP scaffold, further demonstrating the successful loading of BP onto the surface of the PCL scaffold (Figure S2B, Supporting Information).

Owing to the extended duration required for bone repair processes, it is important to develop bone repair materials that have a controlled release and remain in the body for a certain period. Therefore, the detection of the released BP by PCP, which has a BP concentration of 21.2 ± 0.3 ng mm^−2^, as well as the degradation of BP, were recorded. We discovered that BP was gradually released from PCP over time, requiring ≈12 days for complete release. In agreement with this, the surface color of the PCP also changed from brown–yellow to white (Figures 1C,D). In addition, the degradation profile of BP in PCP was examined in vivo by implanting PCP into the skin. Within the PCP, the BP was labeled with CY7 fluorescence and emitted red light when activated by infrared light. As shown in Figure 1E, the subcutaneous fluorescence intensity gradually decreased over time and disappeared after 11 days. These results indicate that BP in the PCP scaffolds gradually degrades in the body and can persist for ≈11 days (Figure 1F). Collectively, these data indicate that PCP exhibits slow BP release and degradation.

### 2.2 Biocompatibility Properties of PCP Scaffolds

**Figure 2:**
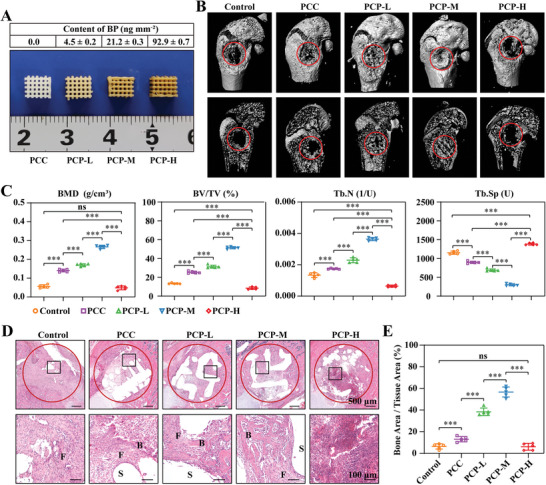
Dose‐dependent evaluation of BP in bone regeneration. A) Photographs of the PCC and PCP scaffolds loaded with different doses of BP. B) Micro‐CT 3D reconstruction images of the bone defect after scaffold implantation for 4 weeks. C) Bone trabecular analysis parameters, including BV/TV, BMD, Tb.N, and Tb.Sp in the bone defect 4 weeks after the implantation of the scaffolds, *n* = 5. D) HE staining of the bone defects after 4 weeks of scaffold implantation. F, fibrous tissue; B, bone tissue; S, scaffold. E) The percentage of bone area at the bone defect to tissue area. All data presented as mean ± SEM and *p*‐values were analyzed by one‐way ANOVA in C and E. ns, with no significant difference, ^***^ *p* < 0.001. All data are representative of two to three independent experiments.

Considering the potential toxicity of BP nanosheets,^[^ ^25^, ^33^ ^]^ the use of high doses could result in unfavorable side effects. To address this concern, PCP scaffolds with low (PCP‐L, 4.5 ± 0.2 ng mm^−2^), medium (PCP‐M, 21.2 ± 0.3 ng mm^−2^), and high (PCP‐H, 92.9 ± 0.7 ng mm^−2^) doses of BP were applied to investigate the dose‐dependent effects of BP, respectively (**Figure** **2**A). As shown in Figure 2B, PCP‐L and PCP‐M significantly promoted bone regeneration, with PCP‐M demonstrating a notably superior effect compared with PCP‐L. However, PCP‐H clearly inhibited new bone formation, with the amount of new bone formation in the PCP‐H group being lower than that in the control group without scaffolds. This was demonstrated by analyzing bone mineral density (BMD), cancellous bone volume/tissue volume (BV/TV), trabecular number (Tb.N), and trabecular separation (Tb.Sp) using micro‐quantitative computed tomography (micro‐CT) (Figure 2C). Hematoxylin and eosin (HE) staining was conducted to evaluate the pathological changes at the injury sites 4 weeks after scaffold implantation in each group. The HE‐stained sections indicated the presence of newly formed bone, fibrous tissue, and scaffolds (Figure 2D). In the groups that underwent PCC, PCP‐L, and PCP‐M scaffold implantation, it was observed that the latticing scaffold effectively filled the defect area, with the spaces between the scaffold struts being filled by newly formed tissues. Additionally, mineralized bone tissue was observed along the surface of the struts (Figure 2D). However, in the control and PCP‐H groups, the defect was not fully filled and only a few scattered fibrous tissues were present. Although both the PCP‐L and PCP‐M groups showed increased amounts of new bone tissue, the PCP‐M group demonstrated a higher level of new bone tissue ingrowth than the PCP‐L group (Figure 2E).

**Figure 3:**
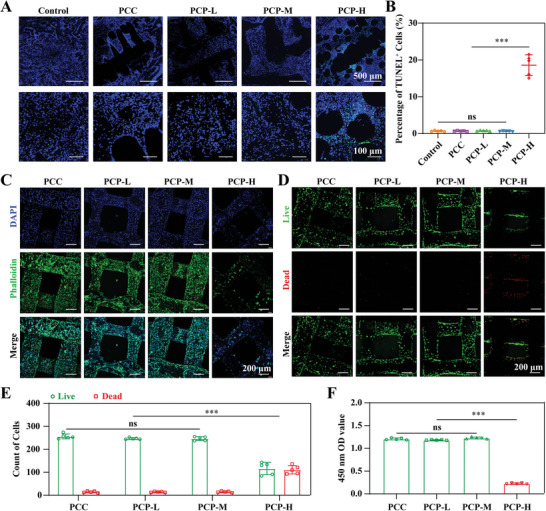
Biocompatibility properties of PCP scaffolds. A) TUNEL staining of bone defects after 4 weeks of scaffold implantation. B) Proportion of TUNEL^+^ cells in bone defects. C) Phalloidin/DAPI staining of BMSCs 48 h after seeding on the scaffold surface. D) Live/Dead staining of BMSCs 48 h after seeding on the scaffold surface. E) Count of live and dead cells, *n* = 5. F) CCK8 assay after seeding BMSCs on the scaffold surface for 48 h, *n* = 5. All data presented as mean ± SEM and *p*‐values were analyzed by one‐way ANOVA in B and F, and two‐way ANOVA in E. ns, with no significant difference, ^***^ *p* < 0.001. All data are representative of two to three independent experiments.

Terminal deoxynucleotidyl transferase dUTP nick‐end labeling (TUNEL) staining was used to detect apoptosis in each group. The TUNEL staining results confirmed the presence of a significant number of dead cells around the skeleton in the PCP‐H group, indicating that the concentration of BP on the PCL scaffold significantly hampered the cell viability when it reaches 92.9 ± 0.7 ng mm^−2^ (**Figure** **3**A). This could account for the impaired healing of bone defects observed in the PCP‐H scaffold group. In addition, we conducted in vitro biocompatibility studies. Cytoskeletal staining of BMSCs with phalloidin showed good adhesion and extension of cells along the surfaces of PCP‐L and PCP‐M, whereas PCP‐H significantly inhibited the proliferation of BMSCs (Figure 3C). Live/dead staining consistently revealed that most of the seeded cells remained alive on PCP‐L and PCP‐M scaffolds, with only a few dead cells being observed. In contrast, only a few cells remained alive on the PCP‐H scaffolds, and most of the seeded cells died (Figure 3D,E). Moreover, the Cell Counting Kit‐8 (CCK‐8) assay demonstrated that cell viability and proliferation activity were comparable among the PCC, PCP‐L, and PCP‐M groups. However, cell viability in the PCP‐H group was approximately one‐sixth that in the other three groups (Figure 3F).

To further determine whether the local application of PCP scaffolds would cause systemic toxic effects in vivo, blood biochemical indicators and the histopathology of major organs were examined. The results depicted in Figure S3A–C (Supporting Information) demonstrate that the implantation of PCP‐L, PCP‐M, and PCP‐H into the bone‐defect site for 1 or 2 weeks did not result in significant changes in the serum concentrations of albumin, alkaline phosphatase, alanine aminotransferase, aspartate aminotransferase, urea nitrogen, creatinine, creatine kinase, and lactate dehydrogenase. These indicators commonly reflect the functions of the heart, liver, and kidneys in clinical settings. Furthermore, as shown in Figure S4 (Supporting Information), there was no obvious damage or abnormal histological changes in the heart, liver, spleen, lungs, brain, or kidneys of rats in the PCP‐L, PCP‐M, and PCP‐H groups 1 or 2 weeks after implantation. In conclusion, these results indicate that the local application of PCP scaffolds does not exhibit systematic toxic effects in vivo.

Therefore, the above results confirm that PCP‐L and PCP‐M exhibit good biocompatibility both in vivo and in vitro. Given that PCP‐M demonstrated a more pronounced ability to promote bone regeneration than PCP‐L, it was selected for further investigation in this study and will hereafter be referred to as PCP.

### 2.3 PCP Scaffold Accelerates Bone Regeneration

To verify the ability of the PCP scaffolds with respect to accelerating bone regeneration, a 3‐millimeter diameter bone defect was created in the femoral condyle of the rats using a drill (Scheme 1C; Video S1, Supporting Information). The integration of PCL composites with a new 3D printing method is a promising approach for the effective treatment of bone injuries.^[^ ^34^ ^]^ Besides providing mechanical support to the bone defect site, the porous structure of the printed PCL scaffold can also offer space for cell accommodation and migration and facilitate nutrient exchange between the scaffold and the environment.^[^ ^34^ ^]^ Furthermore, we discovered that PCP can effectively sustain the release of BP, thus preventing the burst release that can occur with the local application of a BP solution. Therefore, PCP implantation was used instead of BP solution injection to repair bone defects.

**Figure 4:**
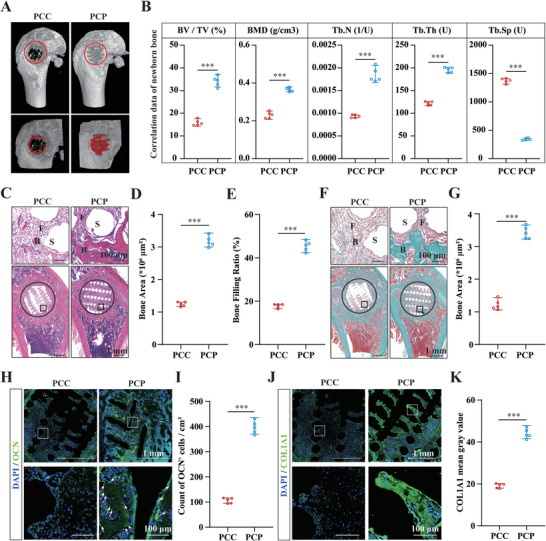
PCP scaffold accelerates bone regeneration. A) Micro‐CT three‐dimensional reconstruction images of the bone defect after scaffold implantation for 4 weeks. B) Bone trabecular analysis parameters, including BV/TV, BMD, Tb.N, Tb.Th, and Tb.Sp in the bone defect 4 weeks after the implantation of the scaffolds, *n* = 5. C) Representative images of HE staining at the bone defect site. B, bone tissue. F, fibrous tissue. S, scaffold. D) Newly formed bone area quantified based on HE staining, *n* = 5. E) Percentage of scaffold pores filled with newly formed bone, *n* = 5. F) Representative images of Masson staining at the bone defect site. B, bone tissue. F, fibrous tissue. S, scaffold. G) Newly formed bone area quantified based on Masson staining, *n* = 5. H) Representative images of OCN IF staining at the bone defect site, with white arrows indicating OCN^+^ osteoblasts. I) The count of OCN^+^ osteoblasts at the bone defect site, *n* = 5. J) Representative images of COL1A1 IF staining at the bone defect site. K) The mean gray value of COL1A1 IF staining, *n* = 5. All data presented as mean ± SEM and *P* values were analyzed by two‐tailed *t*‐tests in B, D, E, G, I, and K. ***P < 0.001. All data are representative of two to three independent experiments.

In addition, facilitated bone formation was demonstrated by micro‐CT analysis (**Figure** **4**A,B). Hematoxylin and eosin (HE) staining showed that almost all scaffold pores in the bone defect of the PCP group were filled with new bone (Figure 4C), as confirmed by the increased bone area and scaffold bone‐filling ratio (Figure 4D,E). Masson staining revealed that most of the newly formed bone was mature calcified bone in the PCP group, whereas the PCC group had only mature bone at the periphery and mainly fibrous tissue in the central part of the bone defect (Figure 4F), as confirmed by the increased mature calcified bone area in the bone defect (Figure 4G), which suggests that BP may promote the deposition of minerals and accelerate bone maturation.

The differentiation of BMSCs into osteoblasts was detected by tissue immunofluorescence (IF) staining. As an osteoblast‐specific marker, osteocalcin (OCN) was used to identify osteoblasts.^[^ ^35^ ^]^ IF staining revealed a remarkable increase in the number of OCN‐positive (OCN^+^) osteoblasts in the PCP group compared to those in the PCC group (Figure 4H,I). Consistently, the expression of collagen I (COL1A1), the primary extracellular organic matter synthesized and secreted by osteoblasts,^[^ ^36^ ^]^ was significantly higher in the PCP group than in the PCC group (Figure 4J). This was demonstrated by the mean grey value of COL1A1 IF staining (Figure 4K). In conclusion, these data confirm that PCP scaffolds significantly accelerate bone regeneration and maturation compared with PCC scaffolds.

### 2.4 Transcriptomic Mechanisms of Signal Transduction and Regulatory Networks of BP in Bone Regeneration

**Figure 5:**
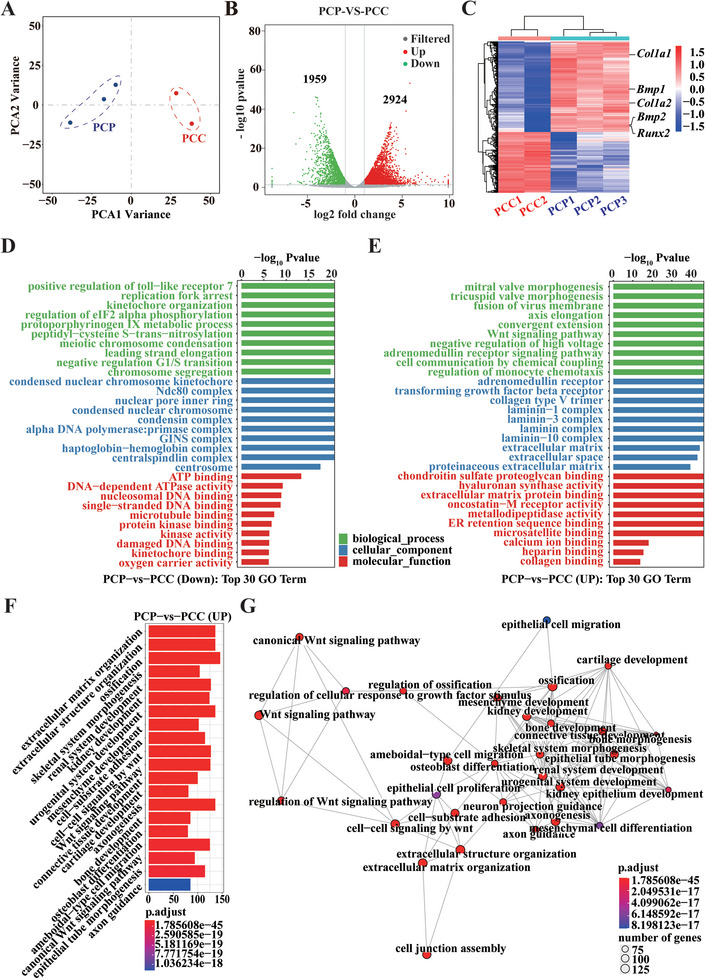
Differential gene expression between PCC and PCP groups was analyzed by RNA‐seq one week after scaffold implantation into the bone defect. A) PCA analysis of all samples. B) The DEGs between the PCC and PCP group. C) Heatmap analysis of DEGs. D) The top 30 up enrichment GO terms in the PCP group compared to the PCC group. E) The top 30 down enrichment GO terms in the PCP group compared to PCC groups. F) Up enrichment GO terms related to the bone in the PCP group compared to the PCC group. G) A network diagram of interactions between bone regeneration‐related biological processes in differentially expressed genes.

To clarify how BP regulates gene expression and network regulation of local tissue cells during bone regeneration. The scaffold was obtained one week after implantation along with the tissue cells that grew into it, and RNA‐seq was performed (Scheme 1C,D). Statistical analysis of the samples in the two groups was performed by principal component analysis (PCA), and the results showed that the transcriptome data could be used for further analysis (**Figure** **5**A). The correlation coefficients between sequenced samples were obtained based on gene expression, and the results showed a good correlation within groups and a relatively low correlation between groups (Figure S5A, Supporting Information). Sample‐to‐sample clustering analysis also showed the same results (Figure S5B, Supporting Information), which indicated slight differences within groups and significant differences between groups. Differentially expressed genes (DEGs) were then analyzed, and there were 2924 genes up‐regulated (>1.5‐fold, *p* < 0.05) and 1959 genes down‐regulated (<0.66‐fold, *p* < 0.05) in the PCP group compared to those in the PCC group (Figure 5B; Figure S5C, Supporting Information). The heatmap revealed the expression pattern of the DEGs (Figure 5C), which was consistent with previous reports.^[^ ^37^ ^]^ several leading osteogenic differentiation‐related genes are highly expressed, including *Col‐1*, runt‐related transcription factor 2 (*Runx2)*, and bone morphogenetic protein 2 (*Bmp2)*.

Gene ontology (GO) enrichment was used to analyze the functions of the DEGs at the biological process, cellular component, and molecular function levels. The top 30 upregulated GO terms were used to compare PCP and PCC groups. Among the downregulated terms, biological processes primarily associated with mitosis included replication fork arrest, kinetochore organization, meiotic chromosome condensation, leading strand elongation, regulation of the G1/S transition, and chromosome segregation (Figure 5D). The cellular components participating in these biological processes include condensed nuclear chromosome kinetochores, Ndc80 complexes, nuclear pore inner rings, condensed nuclear chromosomes, condensin complexes, primase complexes, GINS complexes, central spindlin complexes, and centrosomes. Molecular functions suggested that the downregulated genes regulated mitosis mainly by affecting ATP binding, DNA‐dependent ATPase activity, DNA binding, protein kinase binding, and kinetochore binding. This mitotic inhibitory function of BP suggests its potential as an antitumor therapy. Consistent with previous reports, BP has been reported to inhibit mitosis and survival of prostate cancer cells.^[^ ^38^ ^]^ In addition to inhibiting mitosis in the downregulated terms, BP inhibited the positive regulation of Toll‐like receptor seven and protoporphyrinogen IX metabolic processes. The activation of Toll‐like receptor seven is thought to rapidly induce the release of cytotoxic cytokines and thus, kill cells,^[^ ^39^ ^]^ suggesting that BP may protect cells by downregulating the release of local deleterious cellular inflammatory factors. Protoporphyrinogen IX metabolism is an essential biological process in heme synthesis,^[^ ^40^ ^]^ and molecular function suggests that BP may lead to a decrease in oxygen transport activity. These results suggest that BP may inhibit cell mitosis, cytokine release, and oxygen transport.

The upregulated biological processes included cardiac valve morphogenesis, Wnt signaling, adrenomedullin signaling, electrical signaling, and monocyte chemotaxis regulation (Figure 5E). Wnt signaling, adrenomedullin signaling, and monocyte chemotaxis regulation play critical roles in bone regeneration.^[^ ^41^ ^]^ These biological processes involve cellular components, such as adrenomedullin receptors, transforming growth factor beta receptors, laminin complexes, and extracellular matrix spaces. Regarding molecular functions, the upregulated genes were mainly involved in regulating extracellular matrix synthesis, including chondroitin sulfate proteoglycan, hyaluronan synthase, extracellular matrix protein, and collagen. All these components are essential components of the bone matrix.^[^ ^42^ ^]^ Among these, hyaluronic acid participates in the axial extension of bones,^[^ ^43^ ^]^ which is also related to biological processes. In addition, the oncostatin‐M receptor and calcium ions play a role in bone modeling and remodeling to promote osteogenesis.^[^ ^44^ ^]^ These results suggested that BP promoted extracellular matrix synthesis during bone regeneration. Furthermore, the wnt signaling pathway, the adrenomedullin signaling pathway, and the transforming growth factor *β* signaling pathway may act as significant signal transduction regulatory pathways. In particular, the transforming growth factor *β* signaling pathway has been considered a critical regulatory pathway in the extracellular matrix regulatory network.^[^ ^45^ ^]^

Osteogenic directional enrichment analysis was performed to further understand the osteogenesis‐related processes regulated by BP. The results showed that BP mainly participated in extracellular matrix organization, extracellular structural organization, ossification, skeletal system morphology, renal system development, cartilage development, osteogenic differentiation of BMSCs, and osteoblast differentiation (Figure 5F). The brain map shows the relationship between the genes involved in these processes in the PCP group of upregulated genes and their corresponding processes (Figure S5D, Supporting Information). The bioprocess interaction map revealed these process interactions and suggested that the Wnt signaling pathway plays a primary regulatory role (Figure 5G).

### 2.5 BP Creates an Osteoimmune‐Friendly Microenvironment at the Bone Injury Site

**Figure 6:**
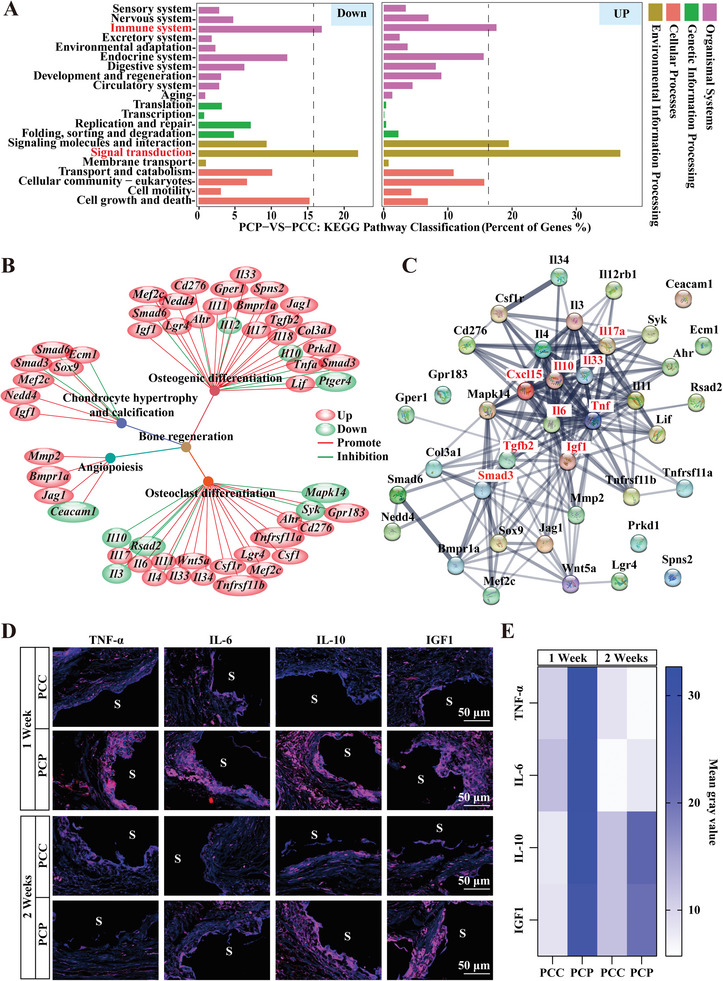
BP regulates immune signaling in bone regeneration. A) A KEGG enrichment analysis of differentially expressed genes between PCP and PCC groups. B) A summary of the expression and role of immune molecules associated with bone regeneration. C) A PPI analysis of the interactions of immune molecules associated with bone regeneration. D) IF staining images of local pro‐inflammatory factors, TNF‐*α* and IL‐6, and anti‐inflammatory factors, IL‐10 and IGF1, at 1 and 2 weeks after PCC and PCP scaffold implantation. S, scaffold. E) Analysis of mean grays value of IF staining, *n*  = 5.

To further understand the BP‐regulated metabolic pathways associated with bone, we used the Kyoto Encyclopedia of Genes and Genomes (KEGG) enrichment analysis to understand the functions of the DEGs. The results showed that among the upregulated and downregulated genes, signal transduction‐related genes occupied the most dominant proportion (**Figure** **6**A), which was expected because the bone repair process involves communication and differentiation between multiple cells, such as vascular endothelial cells and osteoblasts.^[^ ^46^ ^]^ Consistent with previous studies, we found that organismal system entries showed that DEGs were significantly concentrated in the immune system, suggesting that BP has the potential for immune and inflammatory regulation (Figure 6A). Therefore, we further consolidated the immune molecules identified in the DEGs that participated in bone regeneration. The results showed that these immune molecules primarily promoted osteogenic and osteoclastic differentiation, and to a lesser extent, angiogenesis, chondrocyte hypertrophy, and calcification (Figure 6B). Protein–protein interaction networks (PPI) revealed a complex network of interactions for most of these immune molecules and that the IL‐33, Interleukin 17a (IL‐17a), IL‐10, Chemokine (C‐X‐C motif) Ligand 15 (CXCL15), IL‐6, TNF, IGF1, TGF*β*2, and SMAD family member three (SMAD3) may be central to local immune regulation (Figure 6C). Moreover, BP regulated the expression of both the major pro‐inflammatory factors IL‐6 and TNF‐*α* and the major anti‐inflammatory factors IL‐10, IGF1, and TGF‐*β*2 (Figure 6C), suggesting that BP can effectively amplify the benefits of early acute inflammation and promote the expression of anti‐inflammatory factors to facilitate tissue repair.IF staining showed that one week after scaffold implantation, the PCP scaffold significantly promoted the expression of TNF‐*α*, IL‐6, IL‐10, and IGF1 in its surrounding cells. Two weeks after the implantation, the expression of pro‐inflammatory factors, TNF‐*α* and IL‐6 were observed to be decreased; in contrast, anti‐inflammatory factors showed persistently high expression (Figure 6D), as confirmed by the mean gray value analysis of IF staining (Figure 6E). These results suggest that BP initially activates the expression of pro‐inflammatory genes, amplifying the benefits of acute inflammation. Subsequently, it promotes the secretion of anti‐inflammatory factors to accelerate the regression of inflammation. This, in turn, creates a favorable immune microenvironment for bone regeneration.

### 2.6 BP Stimulates IL‐33 Expression in Macrophages

In addition to the major anti‐inflammatory and pro‐inflammatory factors, among the upregulated DEGs, IL‐33 attracted our attention (Figure 6B). IL‐33 is a member of the IL‐1 family of cytokines.^[^ ^47^ ^]^ Under physiological conditions, IL‐33 is highly expressed in various cell types, particularly in epithelial cells, endothelial cells, and fibroblasts.^[^ ^48^ ^]^ IL‐33 acts as an endogenous danger signal or warning signal after cellular injury, alerts the immune system to tissue damage during trauma or infection, and amplifies inflammatory signals in response to tissue damage.^[^ ^49^ ^]^ IL‐33 is thought to be the initial response signal to tissue injury, and amplifies the inflammatory response after tissue injury to recruit various types of cells to initiate tissue repair. Recent studies have found that, in addition to triggering early pro‐inflammatory gene expression, IL‐33 induces rapid metabolic remodeling of macrophages, thus promoting their polarization into alternatively activated macrophages to promote inflammation regression and tissue repair.^[^ ^50^ ^]^ The PPI analysis revealed that IL‐33 regulated the expression of most of the core immune molecules that were upregulated in the PCP group (Figure 6C). This finding aligns with those of previous reports demonstrating that IL‐33 promotes the expression of IL17,^[^ ^51^ ^]^ IL10,^[^ ^52^ ^]^ IL6, TNF,^[^ ^53^ ^]^ and IGF1.^[^ ^54^ ^]^ Furthermore, the IL‐33 signaling axis and the Wnt signaling pathway have a synergistic relationship,^[^ ^55^ ^]^ suggesting an essential role for IL‐33 in BP‐mediated bone regeneration.

**Figure 7:**
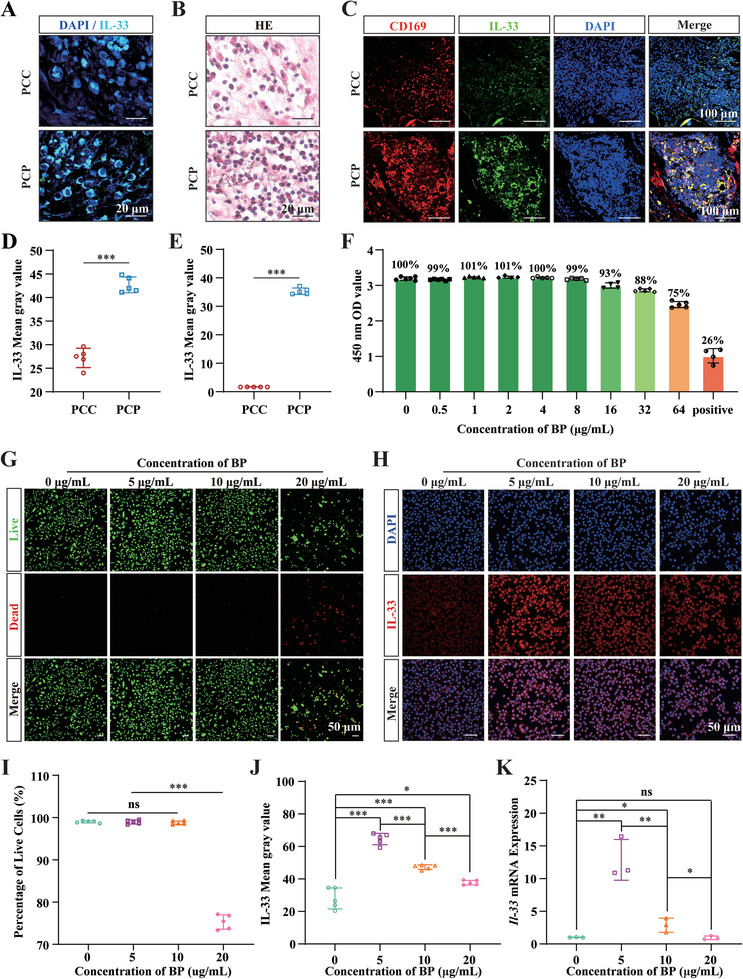
BP stimulates IL‐33 expression in macrophages. A) Representative images of IL‐33 IF staining at one week of PCC and PCP scaffold implantation. B) Representative images of HE staining at one week of PCC and PCP scaffold implantation. C) Representative images of CD169 and IL‐33 IF co‐staining at one week of PCC and PCP scaffold implantation. D,E) Mean gray value analysis of IL‐33 IF staining, *n* = 5. F) Statistical analysis of cell viability after treatment of macrophages with different concentrations of BP, *n* = 5. G) Representative images of live/dead staining after treatment of macrophages with different concentrations of BP. H) Representative images of IL‐33 IF staining after treatment of macrophages with different concentrations of BP. I) A statistical analysis of the percentage of live cells after treatment of macrophages with different concentrations of BP, *n* = 5. J) An analysis of the mean gray value of IL‐33 IF staining, *n* = 5. K) Statistical analysis of *Il‐33* mRNA expression after treatment of macrophages with different concentrations of BP, *n* = 3. All data presented as mean ± SEM and *p*‐values were analyzed by two‐tailed *t*‐tests in (D) and (E) and one‐way ANOVA in (I–K). ns, with no significant difference, ^*^ *p* < 0.05, ^**^ *p* < 0.01, ^***^ *p* < 0.001. All data are representative of two to three independent experiments.

Given the high overlap between the pattern of BP regulation of local inflammatory signaling and IL‐33 regulation of inflammatory signaling and the elevation of IL‐33 in BP‐mediated bone regeneration, we hypothesized that BP creates a beneficial immune microenvironment for bone regeneration by stimulating IL‐33 expression to amplify early inflammatory responses and mediate subsequent inflammatory regression. To test this hypothesis, we initially identified the cell that expressed IL‐33 predominantly after implanting the PCP scaffold. IF staining showed that PCP significantly increased IL‐33 protein expression and the number of IL‐33‐positive cells compared to PCC (**Figure** **7**A,D). HE staining showed that cells with high IL‐33 expression were likely macrophages (Figure 7B). This speculation is consistent with the biological processes by which BP regulates monocyte chemotaxis (Figure 5E). Immunostaining of IL‐33 with the macrophage‐specific marker, CD169, confirmed that PCP primarily induced IL‐33 expression in macrophages (Figure 7C,E).

To further investigate whether BP promotes IL‐33 expression in macrophages in vitro, we first examined the safe application of BP in macrophages. Macrophages were incubated with various concentrations of BP for 24 h. The results demonstrated that BP concentrations exceeding 32 µg mL^−1^ significantly impeded macrophage activity, whereas concentrations below 16 µg mL^−1^ fell within a relatively safe range for application. (Figure 7F). Live/dead staining demonstrated that concentrations <10 µg mL^−1^ is a safe application concentration; in contrast, 20 µg mL^−1^ resulted in significant death of macrophages (Figure 7G), as confirmed by the percentage of live cells (Figure 7I). IL‐33 IF staining revealed that BP stimulated the expression of IL‐33 in macrophages, with the most significant effect observed at a concentration of 5 µg mL^−1^. However, the expression of IL‐33 decreased when the concentration was further increased (Figure 7H), as confirmed by the mean gray values obtained by IL‐33 IF staining (Figure 7J). Consistent results were also obtained when detecting *Il‐33* mRNA expression, with BP at 5 µg mL^−1^ significantly promoting *Il‐33* gene expression in macrophages (Figure 7K). Collectively, these results demonstrated that BP can stimulate the expression of IL‐33 in macrophages.

### 2.7 IL‐33 Promotes Osteogenic Differentiation of BMSCs and Bone Mass Formation

**Figure 8:**
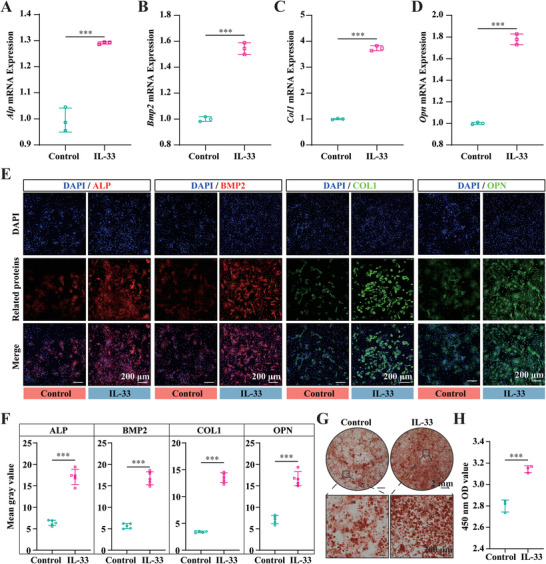
IL‐33 promotes osteogenic differentiation of BMSCs in vitro. A–D) Expression of osteogenic‐related mRNAs, including *Alp* (A), *Bmp2* (B), *Col‐1* (C), and *Opn* (D), after 7 days culture of rat BMSCs with osteogenic induction medium containing 50 ng mL^−1^ IL‐33, *n* = 3. E) Representative images of osteogenic‐related proteins IF staining, including ALP, BMP2, COL‐1, and OPN, after 7 days of culture of rat BMSCs with osteogenic induction medium containing 50 ng mL^−1^ IL‐33. F) The mean gray value of osteogenic‐related proteins IF staining, *n* = 5. G) Representative images of rat BMSCs alizarin red staining after twenty‐one days of culture with osteogenic induction medium containing 50 ng mL^−1^ IL‐33. H) Quantitative analysis of alizarin red staining, *n* = 3. All data presented as mean ± SEM and *P* values were analyzed by two‐tailed *t*‐tests in (A–D), (F), and (H). ^***^ *p* < 0.001. All data are representative of two to three independent experiments.

In addition to modulating the immune response to tissue injury, IL‐33 has recently received much attention in bone remodeling^[^ ^56^ ^]^ as IL‐33 deficiency manifests as accelerated bone loss.^[^ ^57^ ^]^ Having observed that BP promoted the expression of IL‐33 in macrophages, we next sought to determine whether IL‐33 could promote the osteogenic differentiation of BMSCs. Induction of rat BMSCs using an osteogenic induction medium containing 50 ng mL^−1^ IL‐33 resulted in a significant increase in the expression of osteogenesis‐related mRNAs, including alkaline phosphatase (Alp), Bmp2, *Col‐1*, and osteopontin (Opn), after 7 days (**Figure** **8**A–D). Similarly, IF staining demonstrated a significant increase in the corresponding protein expression (Figure 8E), as confirmed by analysis of the mean gray value of IF staining (Figure 8F). Consistent with the histological and RNA‐seq results, IL‐33 significantly promoted mineralization and accelerated calcium salt deposition in the extracellular matrix (Figure 8G), as confirmed by a quantitative analysis of alizarin red staining (Figure 8H). Collectively, these results suggested that IL‐33 significantly promoted the osteogenic differentiation of BMSCs in vitro.

**Figure 9:**
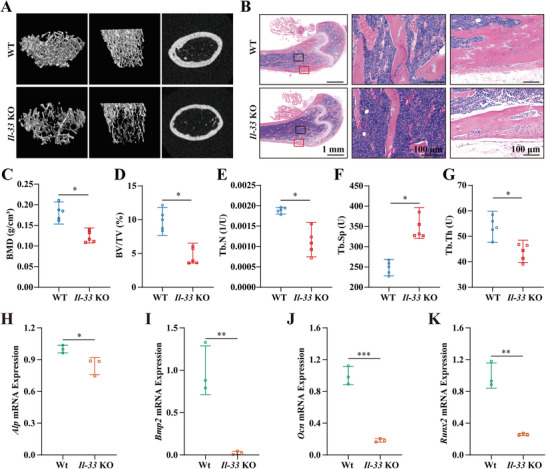
IL‐33 promotes bone mass formation and osteogenesis in vivo. A) Micro‐CT images of the femur of 8‐week‐old *Il‐33* KO mice and their littermate controls. B) HE staining images of the femur of 8‐week‐old *Il‐33* KO mice and their littermate controls. Middle, Bone trabeculae. Right, Bone Cortices. C–G) Bone trabecular analysis parameters, including BMD, BV/TV, Tb.N, Tb.Th, and Tb.Sp in femurs of eight‐week‐old *Il‐33* KO mice and their littermate controls, *n* = 5. H‐K) Osteogenic‐related mRNA expression in the bone marrow of *Il‐33* KO mice and their littermate controls, including *Alp* (H), *Bmp2* (I), *Ocn* (J), and *Runx2* (K), *n* = 3. All data presented as mean ± SEM and *p‐*values were analyzed by two‐tailed *t*‐tests in (C–K). ^*^ *p* < 0.05, ^**^ *p* < 0.01, ^***^ *p* < 0.001. All data are representative of two to three independent experiments.

To further investigate the role of IL‐33 in vivo, osteogenesis and bone mass formation were compared between *Il‐33* knockout (*Il‐33* ^−/−^) mice and their wild‐type (WT) littermates. *Il‐33* gene knockout mice were created by crossing Il‐33^+/−^ mice to create *Il‐33* ^−/−^ mice (*Il‐33* KO mice). For the control group, littermates of *Il‐33* ^+/+^ mice were used (WT mice). The knockout was confirmed through gene identification mapping and the expression of Il‐33 mRNA in the bone (Figure S6, Supporting Information). *Il‐33* KO mice displayed a markedly low bone mass phenotype consistent with previous reports.^[^ ^58^ ^]^ Micro‐CT analyses revealed sparse bone trabeculae and thinning of the bone cortex in *Il‐33* KO mice (**Figure** **9**A), as indicated by a dramatic decrease in BMD, BV/TV, Tb.N, and Tb.Th and an increase in Tb.Sp (Figure 9C–G). HE staining showed consistent results, exhibiting fewer bone trabeculae, thinner bone trabeculae, and thinner bone cortex in *Il‐33* KO mice (Figure 9B). In addition, the expression of bone marrow osteogenesis‐related genes, including *Alp*, *Bmp2*, *Ocn*, and *Runx2*, was significantly reduced by the deletion of *Il‐33* (Figure 9H–K). Taken together, these results demonstrate that IL‐33 is vital for bone mass formation and the osteogenic differentiation of BMSCs.

### 2.8 BP Promotes Osteogenic Differentiation of BMSCs Through Macrophage‐Derived IL‐33

**Figure 10:**
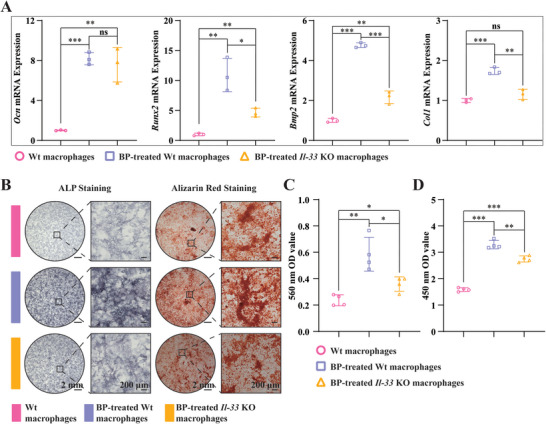
BP promotes osteogenic differentiation of BMSCs through macrophage‐derived IL‐33. A) The expression of osteogenesis‐related genes in BMSCs, including *Ocn*, *Runx2*, *Bmp2*, and *Col‐1* after 7 days of co‐culture with BMSCs using WT bone marrow macrophages, BP‐treated WT bone marrow macrophages, and BP‐treated *Il‐33* KO bone marrow macrophages, respectively, *n* = 3. B) ALP staining after fourteen days of co‐culture with BMSCs and alizarin red staining after twenty‐one days of co‐culture with BMSCs using WT bone marrow macrophages, BP‐treated WT bone marrow macrophages, and BP‐treated *Il‐33* KO bone marrow macrophages, respectively. C) An absorbance analysis of ALP staining at 560 nm, n  = 4. D) Quantitative analysis of alizarin red staining, *n* = 4. All data presented as mean ± SEM and *p*‐values were analyzed by one‐way ANOVA in (A), (C), and (D). ns, with no significant difference, ^*^ *p* < 0.05, ^**^ *p* < 0.01, ^***^ *p* < 0.001. All data are representative of two to three independent experiments.

After confirming that BP promotes IL‐33 expression in macrophages and that IL‐33 promotes the osteogenic differentiation of BMSCs, we sought to determine whether macrophage‐derived IL‐33 is involved in the osteogenic differentiation of BMSCs induced by BP. We cocultured WT macrophages, BP‐treated WT macrophages, and BP‐treated *Il‐33* KO macrophages with WT BMSCs. Osteogenic differentiation of BMSCs was subsequently detected. The results showed that BP‐treated macrophages significantly promoted the expression of genes related to the osteogenic differentiation of BMSCs, including *Ocn*, *Runx2*, *Bmp2*, and *Col‐1* after 7 days of co‐culture, while this promotion effect was significantly decreased after *Il‐33* knockout (**Figure** **10**A). Furthermore, ALP staining after 14 days of co‐culture and alizarin red staining after twenty‐one days of co‐culture showed consistent results (Figure 10B–D). We also observed that knockout of *Il‐33* did not completely eliminate the osteogenic effect of BP‐treated macrophages, suggesting that BP may promote the osteogenic differentiation of BMSCs by stimulating macrophages to express other factors. In conclusion, these results demonstrate that BP promotes the osteogenic differentiation of BMSCs by stimulating the expression of IL‐33 in macrophages.

### 2.9 BP Creates a Pro‐Healing Immuno‐Microenvironment and Accelerates Bone Regeneration Through IL‐33

**Figure 11:**
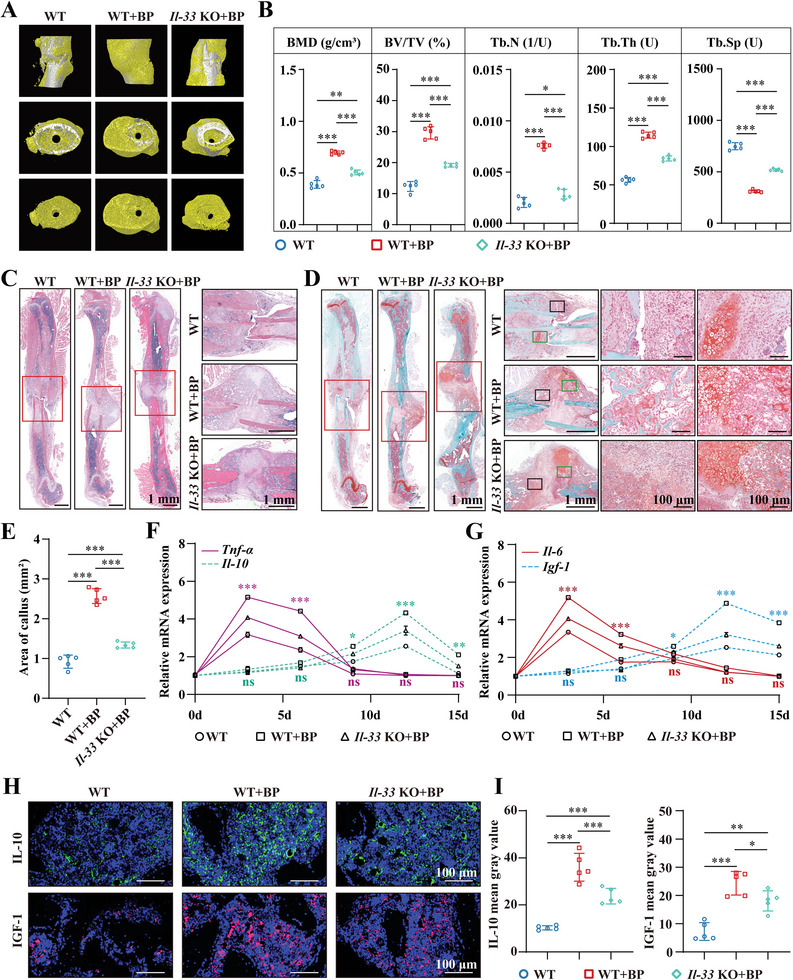
BP creates a pro‐healing immuno‐microenvironment and accelerates bone regeneration through IL‐33. A) Micro‐CT images of WT mice, BP treated WT mice, and BP treated *Il‐33* KO mice 4 weeks after fracture. Yellow, newborn bone. B) Bone trabecular analysis parameters at the fracture site 4 weeks after fracture, including BMD, BV/TV, Tb.N, Tb.Th, and Tb.Sp, n = 5. C) HE staining images of WT mice, BP treated WT mice, and BP treated *Il‐33* KO mice 4 weeks after fracture. D) Safranin O‐Fast Green staining images of WT mice, BP treated WT mice, and BP treated *Il‐33* KO mice 4 weeks after fracture. E) A statistical analysis of the bone callus area, *n* = 5. F, G) Expression profiles of pro‐inflammatory factors, Tnf‐*α* and Il‐6, and anti‐inflammatory factors, Il‐10 and Igf‐1, over time after the administration of BP treatment at femoral fractures in WT and *Il‐33* KO mice*, n* = 5. H) IL‐10 and IGF‐1 IF staining images of WT mice, BP treated WT mice, and BP treated *Il‐33* KO mice 4 weeks after fracture. I) Mean gray value of IL‐10 and IGF‐1 IF staining, *n* = 5. All data presented as mean ± SEM and *p*‐values were analyzed by one‐way ANOVA in B, E, and I, and two‐way ANOVA in (F) and (G). ^*^ *p* < 0.05, ^**^ *p* < 0.01, ^***^ *p* < 0.001. All data are representative of two to three independent experiments.

After observing the enhanced osteogenic immune responses induced by BP and the ability of BP to induce the osteogenic differentiation of BMSCs through IL‐33, we sought to determine whether IL‐33 is involved in the pro‐healing immune microenvironment and accelerates bone regeneration induced by BP. Femoral fractures were induced in *Il‐33* KO mice and their WT littermates, and BP was injected locally into the fracture. Micro‐CT showed that *Il‐33* KO mice had significantly less new bone 4 weeks after fracture than WT mice (**Figure** **11**A). There was also a decrease in the quality of new bone, as evidenced by a dramatic decrease in BMD, BV/TV, Tb.N, and Tb.Th, and an increase in Tb.Sp (Figure 11B). In addition, at 8 weeks after fracture, WT mice showed almost complete remodeling, with only a small amount of immature bone remaining, whereas Il33 KO mice still had more immature bone (Figure S7, Supporting Information). This result is consistent with the histological, RNA‐seq, and in vitro results, suggesting that IL33 may promote mineralization. HE staining showed that a solid external bone callus almost always encapsulated the fracture ends of WT mice, whereas *Il‐33* KO mice, the callus was mainly attached to the fibrous tissue (Figure 11C). Cartilage callus formation is critical for bone regeneration.^[^ ^59^ ^]^ Safranin O‐Fast Green staining showed that the fracture ends of WT mice were completely attached to mineralized bone tissue and cartilage callus, whereas *Il‐33* KO mice showed only a small amount of callus formation (Figure 11D), which was confirmed by the callus area (Figure 11E). These results revealed that BP accelerated bone fracture healing via IL‐33.

In line with the process of bone fracture healing, we found that the expression of pro‐inflammatory cytokines *Il‐6* and *Tnf‐α* rapidly increased in WT mice during the early stages of healing (3 and 6 days) and then decreased at 9 and 12 days (Figures 11F,G). In contrast, the expression of the anti‐inflammatory cytokines *Il‐10* and *Igf‐1* did not significantly increase during the early stages of healing (3 and 6 days), but increased rapidly at 9 and 12 days. However, when *Il‐33* was knocked out, the expression of both pro‐and anti‐inflammatory cytokines induced by BP was significantly reduced. These results suggest that IL‐33 is vital for the osteoimmune response triggered by BP. In addition, IF staining further demonstrated that treatment with BP significantly enhanced the expression of anti‐inflammatory cytokines, including IL‐10 and IGF1, at the injury sites (Figures 11H,I). Nevertheless, the absence of IL‐33 diminished the positive osteoimmune environment. These results show that BP creates a pro‐healing immune microenvironment through IL‐33.

Although these results indicate that BP may promote bone regeneration by facilitating an early acute inflammatory response and subsequent inflammation resolution during the fracture healing process, it is important to note that while enhancing acute inflammation can aid in the recruitment of inflammatory cells to eliminate microorganisms, necrotic tissue, and the provisional fibrin matrix, it also attracts mesenchymal stem cells to initiate bone repair processes.^[^ ^2^, ^26^, ^60^ ^]^ However, it should be emphasized that this process is less predictable and can easily result in excessive acute inflammation, thereby increasing the risk of impaired bone healing, and ultimately hindering bone regeneration.^[^ ^61^ ^]^ Conversely, the failure to resolve inflammation can lead to chronic inflammation, which is detrimental to healing.^[^ ^62^ ^]^ This is why scientists place greater emphasis on the intermediate and late stages of inflammation.

## 3 Conclusion

A suitable regulation of the immune response at the injury site is crucial for creating a microenvironment that promotes bone regeneration and prevents nonunions.^[^ ^2^, ^13^, ^28^ ^]^ Considering the growing significance of BP‐based nanomedicine in promoting bone regeneration, it is essential to thoroughly investigate the immunomodulatory effects of BP nanomaterials at the site of bone injury. The present study shows that BP can accelerate bone regeneration by creating a beneficial immune environment. Initially, the activation of pro‐inflammatory genes by BP enhanced the benefits of acute inflammation. Subsequently, BP promotes the secretion of anti‐inflammatory factors, accelerating the regression of inflammation. As a result, a favorable immune microenvironment for bone regeneration was established. Mechanistically, BP stimulates macrophages to express IL‐33, intensifying the inflammatory response in the early stages, and facilitating the subsequent regression of inflammation. In addition, the expression of IL‐33 in macrophages, mediated by BP, directly promotes the osteogenic differentiation of BMSCs, aiding bone repair. In summary, our study provides evidence supporting the use of BP to accelerate bone regeneration by modulating the immune response at the injury site and transforming it into a regenerative microenvironment. To our knowledge, this is the first time that the immunomodulatory potential of BP in bone regeneration through the regulation of both early‐stage inflammatory response and later‐stage inflammation resolution has been revealed, providing a basis for the clinical application of BP and a potentially effective strategy for regulating the immune microenvironment in bone regeneration.

## 4 Experimental Section

### Fabrication and Characterization of 3D PCL and PCP Scaffolds

PCL powder with a molecular weight of 80 000 was used to fabricate the 3D scaffolds. The scaffolds using an in‐house pneumatic fused‐deposition system mounted to an additive manufacturing device with a nozzle diameter of 200 µm were fabricated. The parameters used for printing were a filament diameter of 200 µm, a filament spacing of 400 µm, and a liquefier temperature of 90 °C. A cellular lattice structure with a 0/90° layered pattern and continuous contour filaments was used to fabricate the 3D porous spatial structure of the scaffold. The scaffolds were first alkalinized to load BP onto the PCL scaffolds using a sodium hydroxide solution (5 mol L^−1^, S111507, Aladdin) for 24 h. Subsequently, the scaffold surface was activated for 12 h using MES buffer (10 mmol mL^−1^, M163014, Aladdin) containing EDC (12 mg mL^−1^, E106172, Aladdin) and NHS (18 mg mL^−1^, H109330, Aladdin), followed by grafting chitosan (1 mg mL^−1^, C434553, Aladdin) onto the activated scaffold surface. BP (200 µg mL^−1^, B463005, Aladdin) was loaded onto the scaffold surface using the principle of interaction between the positive charge on the chitosan surface and the negative charge carried by the oxidized black phosphorus. Scanning electron microscopy (SEM; QUANTA 250, FEI, US) was used to observe whether the BP 2D nanosheets were successfully loaded onto the surface of the PCL scaffold, and the elemental composition of the scaffold surface was analyzed using SEM‐energy spectroscopy (SEM‐EDS, QUANTA 250, FEI, US). For the cellular experiments and in vivo applications, PCL scaffolds grafted with chitosan (PCC) were used in the control group, and PCC scaffolds with BP (PCP) were used in the experimental group.

### BP Loading and Release Experiments

To assess BP loading, PCL scaffolds with known surface areas were loaded with BP and dried. The PCP scaffolds were then dissolved in trichloromethane and the absorbance at 460 nm was measured using a filtered multifunctional enzyme marker (Infinite F200 Pro, Switzerland). For the standard group, a solution containing the same mass percentage of PCL in trichloromethane was used as the solvent to disperse the known mass gradient of BP. The BP loading was calculated from a standard absorbance curve. For in vitro release experiments, the PCP scaffolds were immersed in 0.5 ml deionized water and shaken at 37 °C in a shaker. The soaking solution was collected and replaced daily with fresh deionized water. The phosphate ion concentration in the soaking solution was determined, and the phosphorus content was calculated using a Malachite Green Phosphate Assay Kit (MAK307, Sigma). For in vivo release experiments, Sulfo‐Cyanine7 (CY7) was modified using 6‐mercapto‐1‐hexanol (725 226, Sigma), and the modified CY7 was conjugated to BP to obtain BP‐CY7. PCL scaffolds loaded with BP‐CY7 were implanted in the subcutis of the outer thighs of rats. BP release was assessed every other day by detecting fluorescence intensity using a small‐animal in vivo optical 2D imaging system (IVIS Lumina XR, PerkinElmer).

### In Vitro Biocompatibility

BMSCs were seeded on the surface of the PCP scaffolds and cell viability was detected using the Cell Counting Kit‐8 (CCK‐8) reagent after 48 h. Four hour before the assay, the culture medium was replaced with fresh medium, and 10 µl of CCK‐8 reagent (C0038, Beyotime) was added to each well of 96‐well plates and incubated for 2 h at 37 °C in an incubator, and then 50 µl of incubated culture medium was aspirated into new 96‐well plates and measured at 460 nm by using a filtered multifunctional enzyme marker (Infinite F200 Pro, Switzerland) to calculate cell viability. For live/dead staining, BMSCs were seeded on the surface of the PCP scaffolds for 48 h and incubated for 30 min using the LIVE/DEAD Viability/Cytotoxicity Assay Kit (L32250, Invitrogen). Images were captured using a laser‐scanning confocal microscope (LSCM, ZEISS, Germany). For cytoskeleton staining, BMSCs were seeded on the surface of the PCP scaffolds for 48 h, followed by fixation with 4% paraformaldehyde for 15 min. After staining with phalloidin (F432; Invitrogen) for 1 h, followed by staining with DAPI for 5 min, images were acquired using LSCM.

### In Vivo Biocompatibility

The rats were anesthetized with pentobarbital sodium (50 mg kg^−1^) for 1 and 2 weeks after scaffold implantation, and blood was collected through the orbital venous plexus. After serum separation, the liver, kidney, and cardiac functions were analyzed using a biochemical analyzer (Catalyst Dx, USA). After blood collection, the rats were euthanized using carbon dioxide, and their hearts, livers, spleens, lungs, kidneys, and brains were collected. The tissues were fixed with 4% paraformaldehyde, and 5 µm thick sections were prepared for HE staining. The HE staining protocol is described in detail below.

### Experimental Animals

The experimental procedures, housing, and animal care were approved and performed in accordance with the animal experimentation regulations of the Animal Ethics Committee of Shanghai Jiaotong University (SYXK (hu) 2018‐0027). Sprague‐Dawley (SD) rats and C57BL/6 mice were purchased from Beijing Weitong Lihua Laboratory Animal Technology Co., Ltd. Shanghai Branch (Shanghai, China). The *Il‐33* ^+/−^mice were purchased from Cyagen Biosciences Inc., (Suzhou, China) *Il‐33* ^−/−^ mice and their littermate controls (*Il‐33* ^+/+^) were obtained by the crossbreeding of *Il‐33* ^+/−^ mice and the genotypes were determined by PCR amplification of purified tail genomic DNA using the primers in Tables S1 (Supporting Information). The experimental animals were housed under standard pathogen‐free animal facilities in a temperature‐controlled room (24 ± 2 °C). Preliminary experiments were conducted for all animal studies to determine the sample size requirements. All surgeries were performed under anesthesia, and efforts were made to minimize suffering.

### Rat Bone Defects

Eight‐week‐old male SD rats were used for the bone defect studies. The rats were anesthetized with 2.5% sodium pentobarbital (40 mg kg^−1^ body weight). After sterilization, a sterile towel was laid down and the skin and muscle layers were incised from the thigh to expose the femur. A drill was used to create a 3 mm diameter circular bone defect at the femoral condyle. PCC and PCP scaffolds were implanted, and muscle and skin sutures were performed. Eight rats were used in each group: three for RNA‐seq and five for radiological and histological evaluations.

### Mice Bone Fracture

Eight‐week‐old male *Il‐33* KO mice and their littermate controls were used for the bone fracture studies. After anesthesia with sodium pentobarbital (50 mg kg^−1^), the femur was exposed as described above. A sterile 27‐gauge needle was inserted into the marrow cavity through the articular surface of the proximal femur, temporarily withdrawn to allow mid‐femoral transaction with a scalpel, and then reinserted to stabilize the fracture, as confirmed by radiography. The incision was then closed using sutures. Five mice were used in each group. Starting on postoperative day 2, BP was injected locally into the fracture every 2 days, 20 µl (10 µg ml^−1^) of a suspension of BP in saline was injected each time, and the control group was injected using the same volume of saline. A total of three injections were administered in total. Imaging and histological evaluations were performed 4 weeks postoperatively.

### Micro‐CT

For femoral bone defects in rats at postoperative week 4, for *Il‐33* KO mice at 8 weeks of age, and for fractures in *Il‐33* KO mice at postoperative weeks 4 and 8, the animals were euthanized with CO_2_ and the femur was obtained for micro‐CT (Scanco Medical, Switzerland) evaluation after fixation with 4% paraformaldehyde. The scanning accuracy was set to 10 µm in continuous layers (70 kV and 130 µA radiation source with 0.5 mm aluminum filter). Microstructural analysis of the bone tissue was performed using the Scanco software. Determination of bone mineral density (BMD), BV/TV, Tb.N, Tb.Th, and Tb.Sp was carried out.

### Histological and Immunohistochemical Analysis

For bone histological analysis, femurs were fixed in 4% paraformaldehyde for 48 h, followed by decalcification in 10% EDTA for 4 weeks, then embedded in paraffin wax and stained using 5‐µm‐thick sections for HE staining, Masson staining, Safranin O‐Fast Green staining, and immunohistochemical analysis.^[^ ^63^ ^]^ For HE staining, the sections were dewaxed with water, stained with hematoxylin for 5 min, washed with water, and then treated with 1% hydrochloric alcohol for a few seconds. After rinsing with water, the cytoplasm was stained with eosin for 1 min, dehydrated, and blocked with neutral resin for observation. For Masson's trichrome staining, the prepared thin sections were dewaxed and stained with Weigert iron hematoxylin staining solution (HT1079, Sigma) for 5 min. The sections were differentiated in an acidic ethanol differentiation solution (1% ethanol hydrochloride solution; Sigma–Aldrich) for 10 s and washed with water. The sections were then stained blue in a 1% lithium carbonate aqueous solution (G1841, Solarbio) for 3 min and washed with distilled water for 1 min. Sections were then stained with carmine acidic magenta staining buffer (LZ‐12686, Guidechem) for 5 min and washed with a weak acid working solution (0.3% acetic acid solution, Sigma) for 1 min. The sections were washed with a phosphomolybdic acid solution (20 wt.% ethanol solution, 319 279, Sigma) for 2 min and with a weak acid working solution for 1 min and put into aniline blue staining solution (2.5% acetic acid solution, B8563, Sigma) for 2 min. Subsequently, the sections were washed with weakly acidic working solution for 1 min After dehydration, the sections were sealed with a neutral resin. Safranin O‐Fast Green staining was performed according to the manufacturer's instructions (GP1051; Service Bio). Briefly, the sections were dewaxed in water and stained with Safranin O, followed by Fast Green staining. After dewatering, the sections were sealed with a neutral resin. For immunostaining, the tissue sections were dewaxed in water and the cultured cells were fixed with 4% paraformaldehyde for 10 min and washed three times with PBS. Subsequently, the samples were permeabilized in 0.3% triton X‐100 for 10 min, blocked in 5% goat serum for 30 min at room temperature, and probed with primary antibodies diluted in 5% goat serum in PBS overnight at 4 °C. The following primary antibodies were used: OCN (1:200, MAB1419, R&D), COL‐1 (1:200, ab270993, Abcam), TNF‐*α* (1:200, ab205587, Abcam), IL‐6 (1:200, AF506, R&D), IL‐10 (1:200, AF519, R&D), IGF‐1 (1:200, NBP2‐44968, NOVUS), IL‐33 (1:200, ab187060, Abcam), CD169 (1:200, ab312840, Abcam), ALP (1:200, NB110‐3638, NOVUS), and BMP2 (1:200, ab284387, Abcam). After incubation with the primary antibody, sections were washed with PBS and incubated with the appropriate Alexa Fluor‐coupled secondary antibody for 1 h at room temperature. The cell nuclei were stained with DAPI (; D9542; Sigma). Finally, the sections were analyzed using LSCM. The signal intensity was quantified using ImageJ software.

### Cell

Eight‐week‐old rats and mice were used to extract mononuclear cells and BMSCs. After euthanasia of the rats or mice, the femurs and tibias were obtained and the bone marrow was flushed out using sterile PBS. After dispersing the bone marrow, erythrocytes were lysed using an erythrocyte lysis solution (C3702, Beyotime) and washed with PBS. The obtained bone marrow cells were cultured using Minimum Essential Medium *α* (MEM *α*, 12 571 048, Gibco) containing 10% fetal bovine serum (10 099 141, Gibco) and 1% penicillin‐streptomycin (15 140 148, Gibco) for 24 h. After 24 h, the upper nonadherent cells were cultured for 3 days using MEM *α* containing 50 ng mL^−1^ macrophage colony‐stimulating factor to obtain macrophages. The lower layer of adherent cells was cultured for 7 days and later passaged to the third generation to obtain BMSCs.

### Co‐Culture Experiment

A transwell culture system was used for the co‐culture experiments. BMSCs were inoculated in petri dishes and cultured using a complete culture medium containing 50 µg mL^−1^ ascorbic acid, 10 mm *β*‐glycerol phosphate, and 10^−8^ m dexamethasone. Monocytes were inoculated in Trans‐well chambers and cultured using a complete culture medium containing 50 ng mL^−1^ macrophage colony‐stimulating factor and 5 µg mL^−1^ BP. The mRNA was collected to detect osteogenesis‐related gene expression after 7 days of co‐culture, ALP staining was performed after 14 days of co‐culture, and Alizarin Red staining was performed after 21 days of co‐culture.

### ALP Staining and Alizarin Red Staining

A complete culture medium containing 50 µg mL^−1^ ascorbic acid, 10 mm *β*‐glycerol phosphate, and 10^−8^ m dexamethasone was used for studying the osteogenic differentiation of BMSCs. ALP staining was performed 14 day after induction. The cells were fixed with 4% paraformaldehyde solution and washed three times with phosphate‐buffered saline (P0321S, Beyotime) was used for ALP staining. After incubation for 20 min at room temperature, the cells were washed with PBS. The samples were air‐dried and images were acquired using a body microscope (ZEISS, Germany). The absorbance was measured at 560 nm to quantify the ALP staining. Alizarin Red staining was performed after 21 days after induction. The cells were fixed with 4% paraformaldehyde solution and washed with distilled water to remove salt residues. A solution of Alizarin Red (2 w/v.%, A506786‐0026, Sangon Biotech) with a pH adjusted to 4.2 was added to cover the entire surface of the wells. After incubation for 20 min at room temperature, excess Alizarin Red was washed off with water. The samples were air‐dried and images were acquired using a body microscope. Extraction was performed to quantify the orange–red color of alizarin red by adding a 70% ethanol solution of 10 mM HCl to the stained Petri dishes and incubating for 30 min. Absorbance of the extract was measured at 450 nm.

### QRT‐PCR

Total RNA from BMSCs and macrophages was extracted using TRIzol Reagent (15 596 026, Ambion). The relative RNA expression levels were evaluated using qRT‐PCR, and the housekeeping gene *Actin* was used as a loading control. All PCR amplifications were performed in a final reaction mixture (20.0 µL), and the relative primer sequences were listed in Table S2 (Supporting Information). The amplification reaction was performed using TB Green Premix Ex Taq (RR420A, Takara) for 40 cycles, and relative expression was calculated according to the 2^−ΔΔCt^ method.

### The Whole Transcriptome Analysis

RNA extraction, mRNA library construction, and sequencing were performed as previously reported.^[^ ^64^ ^]^ After the final transcriptome was generated, String‐Tie and ballgown were used to estimate the expression levels of all transcripts and genes by calculating FPKM (FPKM = [total_exon_fragments/mapped_reads (million) × exon_length (kB)]). Differentially expressed transcripts and genes with a fold change >1.5 or fold change <0.66 and *p*‐value <0.05, were selected using the *R* package edgeR.

### Statistical Analysis

All studies were evaluated in at least three independent experiments for each condition to ensure reproducibility, and all experiments were pre‐experimented to determine the sample size. The data were expressed as mean ± SD. Significant differences between different groups were determined using two‐tailed unpaired Student's *t*‐tests for two‐group comparisons and one‐ or two‐way analysis of variance (ANOVA) with post‐hoc Tukey's test for multiple‐group comparisons. For all statistical tests, significance was defined as *P* ≤ 0.05. Statistical analyses were performed using GraphPad Prism software.

## Conflict of Interest

The authors declare no conflict of interest.

## Supporting information

Supporting Information

Supplemental Video 1

## Data Availability

The data that support the findings of this study are available from the corresponding author upon reasonable request.
