# Supplementary material for: Black Phosphorus Accelerates Bone Regeneration Based on Immunoregulation
Source: Adv Sci (Weinh). 2023 Nov 12;11(1):2304824. doi: 10.1002/advs.202304824 (PMC10767454; doi:10.1002/advs.202304824)
Supplement: Supplementary file 1 — Supporting Information [file ADVS-11-2304824-s002.pdf]

ADVANCED SCIENCE
Open Access

Supporting Information

for *Adv. Sci.*, DOI 10.1002/advs.202304824

Black Phosphorus Accelerates Bone Regeneration Based on Immunoregulation

Minglong Qiu, Nijiati Tulufu, Guoqing Tang, Wenkai Ye, Jin Qi, Lianfu Deng* and Changwei Li**

Black Phosphorus Accelerates Bone Regeneration Based on Immunoregulation

Minglong Qiu[#1], *Nijiati Tulufu*[#1], *Guoqing Tang*[#2], *Wenkai Ye*[1], *Jin Qi**[1], *Lianfu Deng**[1], *Changwei Li**[1]

[1]Department of Orthopaedics, Shanghai Key Laboratory for Prevention and Treatment of Bone and Joint Diseases, Shanghai Institute of Traumatology and Orthopaedics, Ruijin Hospital, Shanghai Jiao Tong University School of Medicine, 197 Ruijin 2nd Road, Shanghai 200025, P. R. China.

[2]Kunshan Hospital of Traditional Chinese Medicine, Affiliated Hospital of Yangzhou University, 388 Zuchongzhi Road, Kunshan City, Jiangsu Province.

[#] These authors contributed equally to this work.

*Corresponding authors:

Email addresses: qj40347@rjh.com.cn (*Jin Qi*), lfdeng@shsmu.edu.cn (*Lianfu Deng*), lcw11876@rjh.com.cn (*Changwei Li*)

Keywords: Black phosphorus; bone regeneration; immunoregulation; RNA sequencing; gene knocking-out

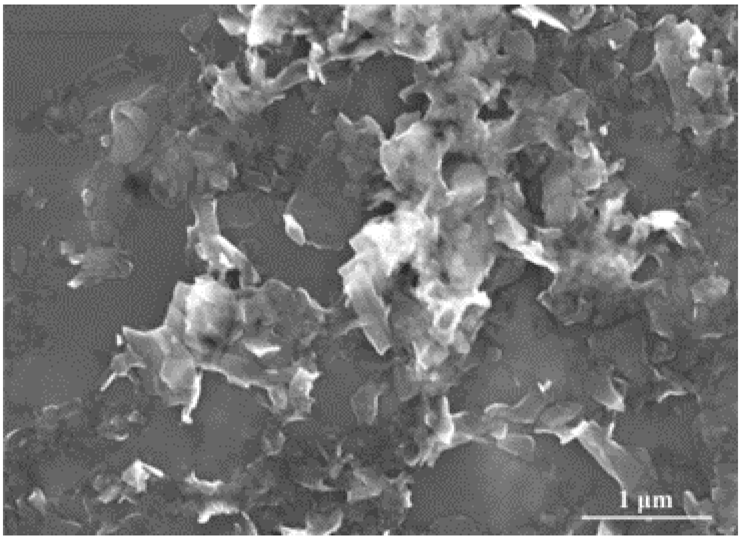

Figure S1. Representative SEM images of two-dimensional black phosphorus nanosheets.

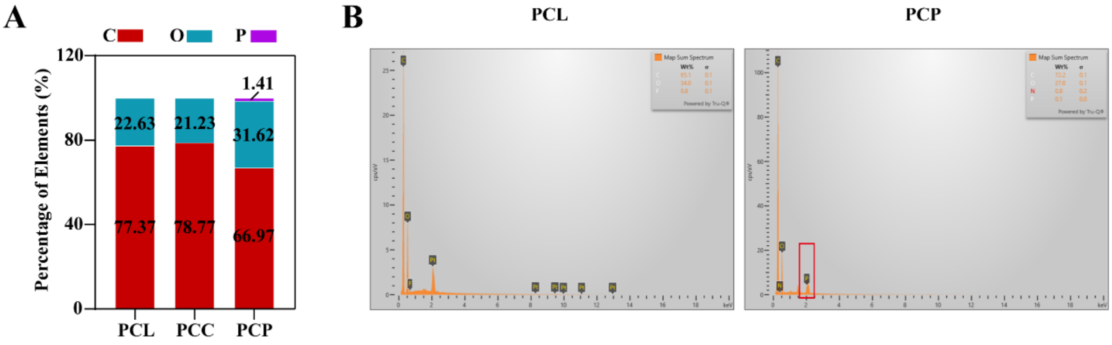

Figure S2. Surface energy spectroscopy of PCL and PCP scaffolds.

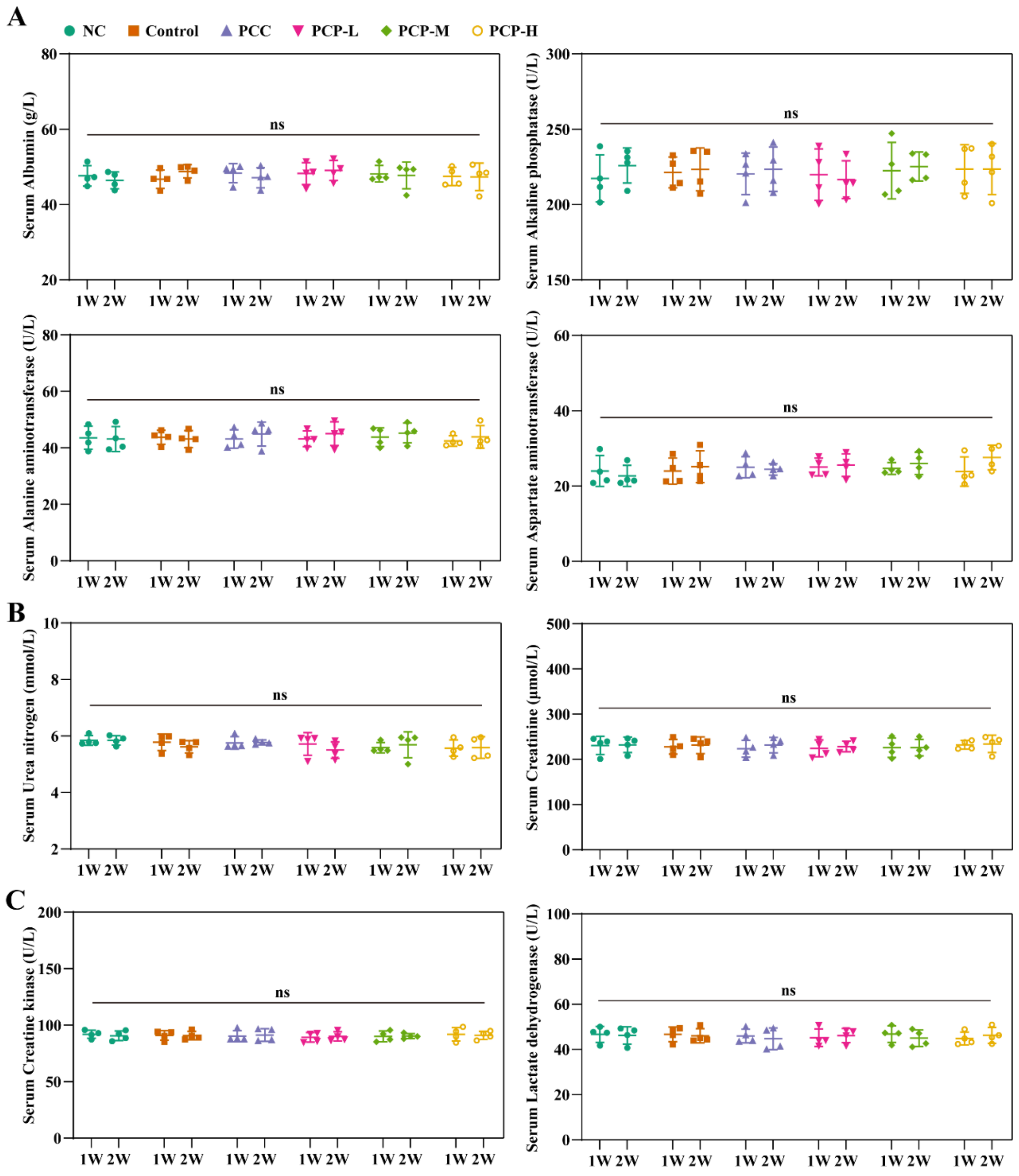

Figure S3. Blood biochemical analysis one and two weeks after scaffold implantation in rat femurs. A) Liver function analysis included serum albumin, alkaline phosphatase, alanine aminotransferase, and aspartate aminotransferase. B) Renal function analysis included serum urea nitrogen and creatinine. C) Cardiac function analysis includes creatine kinase and lactate dehydrogenase. All data presented as mean ± SEM and P values were analyzed by two-way ANOVA in A-C. ns, with no significant difference. All data are representative of two to three independent experiments.

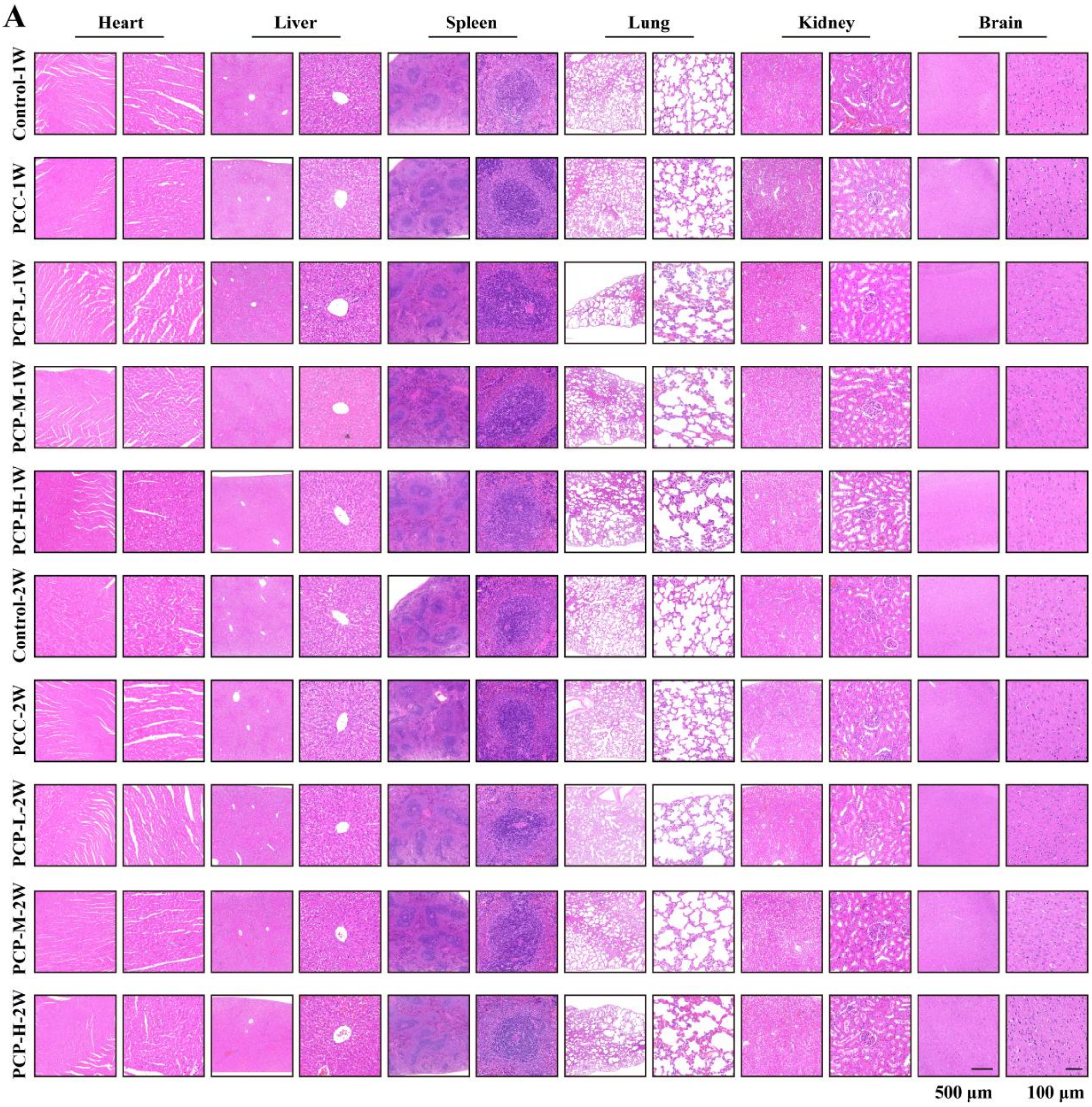

Figure S4. HE staining of heart, liver, spleen, lungs, kidneys, and brain tissues one and two weeks after scaffold implantation in rat femurs.

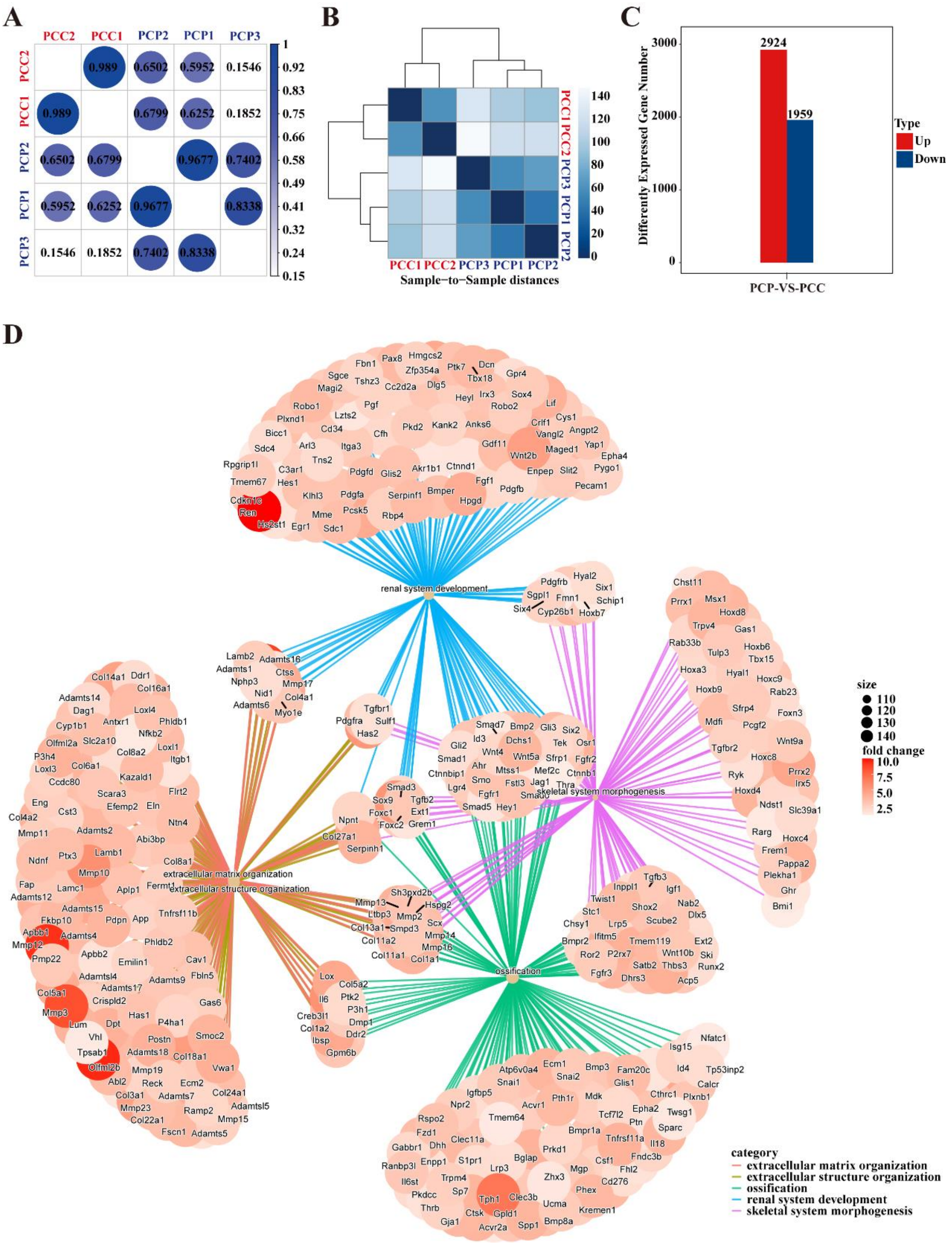

Figure S5. Differential gene expression between PCL and PCP groups was analyzed by RNA-seq two weeks after scaffold implantation into the bone defect. **A) C**orrelation coefficients analysis of all samples. **B)** sample-to-Sample clustering analysis of all samples. **C)** Number of differentially expressed genes in the PCP group compared to the PCL group**. D)** Brain map analysis shows that differentially expressed genes related to bone regeneration are involved in biological processes.

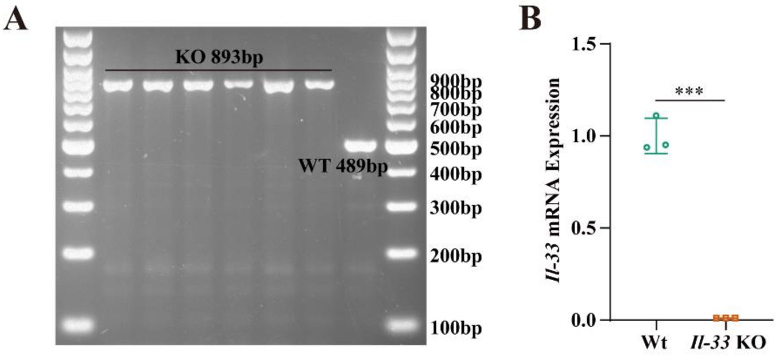

Figure S6. Genetic identification of *Il*-33 KO mice and their littermate controls. A) Genotypic gel electropherograms of *Il-33* KO mice and their littermate controls. B) *Il-33* mRNA expression in bone tissue of eight-week-old *Il-33* KO mice and their littermate controls, $n = 3$. Data presented as mean ± SEM and P values was analyzed by two-tailed t-tests in B. ***$P < 0.001$. All data are representative of two to three independent experiments.

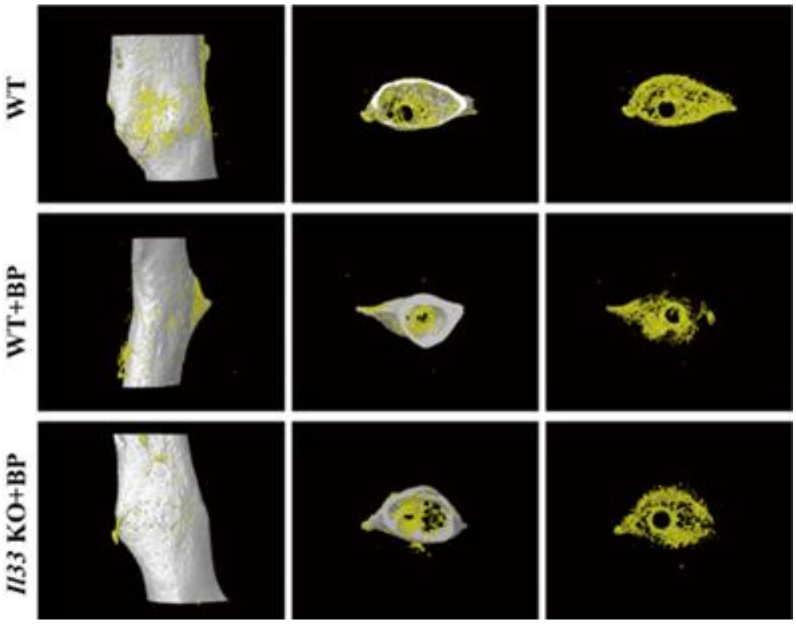

Figure S7. Micro-CT images of WT mice, BP treated WT mice, and BP treated *Il-33* KO mice eight weeks after fracture.

Table S1. Genotyping primers for *Il-33* KO mice

Primer name	Primer sequence（5’→3’）	PCR product size
ex2-Forward	CACTAAGACTACTCAGCCTCAG	

WT-Reverse	CGGTGATGCTGTGAAGTCTG	WT: 489 bp
KO-Reverse	GTGTTCTGCTGGTAGTGGTCG	KO: 893 bp

Table S2. Primer sequences for real time-PCR

Gene		Primer sequence（5’- 3’）	Species
β-actin	Forward	GCTGCACCCTTCAACAACTG	Rat
	Reverse	GTGATCCCGGTCAAAGTGGT	
Il-33	Forward	GCAGCTGCAAGGAAGGGAGAAATCA	Rat
	Reverse	TGGTGAGGCCAGAACGGAGCC	
Alp	Forward	CCCAAGAGACCTTGAAAAATGCCCTGA	Rat
	Reverse	TGTCACTGTGGAGACGCCCATACCAT	
Bmp2	Forward	ATGGTGGCCGGGACCCGCTGT	Rat
	Reverse	CGGGACAAGGGGCGGCCGGA	
Col1	Forward	CCAACGACGTCGAACTTGTTGCTGA	Rat
	Reverse	CAATGTCAAGGAATGGCAGGCGAGA	
Opn	Forward	TTGGCTGAAGCCTGACCCATCTCA	Rat
	Reverse	TCGTCATCATCGTCCATGTGGTCA	
β-actin	Forward	GGCTGTATTCCCCTCCATCG	Mouse
	Reverse	CCAGTTGGTAACAATGCCATGT	
Il-33	Forward	ATGGGAAGAAGCTGATGGTG	Mouse
	Reverse	CCGAGGACTTTTTGTGAAGG	
Alp	Forward	CCAACTCTTTTGTGCCAGAGA	Mouse
	Reverse	GGCTACATTGGTGTTGAGCTTTT	
Bmp2	Forward	GGGACCCGCTGTCTTCTAGT	Mouse
	Reverse	TCAACTCAAATTCGCTGAGGAC	
Ocn	Forward	CTGACCTCACAGATCCCAAGC	Mouse
	Reverse	TGGTCTGATAGCTCGTCACAAG	
Runx2	Forward	ATGCTTCATTCGCCTCACAAA	Mouse

	Reverse	GCACTCACTGACTCGGTTGG	
Col1	Forward	GCTCCTCTTAGGGGCCACT	Mouse
	Reverse	CCACGTCTCACCATTGGGG	
Tnf-α	Forward	GACGTGGAACTGGCAGAAGAG	Mouse
	Reverse	TTGGTGGTTTGTGAGTGTGAG	
Il-10	Forward	GCTCTTACTGACTGGCATGAG	Mouse
	Reverse	CGCAGCTCTAGGAGCATGTG	
Il-6	Forward	TAGTCCTTCCTACCCCAATTTCC	Mouse
	Reverse	TTGGTCCTTAGCCACTCCTTC	
Igf-1	Forward	CTGGACCAGAGACCCTTTGC	Mouse
	Reverse	GGACGGGGACTTCTGAGTCTT	
